# Chemoprevention of Breast Cancer by Dietary Polyphenols

**DOI:** 10.3390/molecules201219864

**Published:** 2015-12-17

**Authors:** Maria-Magdalena Mocanu, Péter Nagy, János Szöllősi

**Affiliations:** 1 Department of Biophysics, “Carol Davila” University of Medicine and Pharmacy, 050474 Bucharest, Romania; mocanu.umfcd@gmail.com; 2 Department of Biophysics and Cell Biology, Faculty of Medicine, University of Debrecen, 4032 Debrecen, Hungary; nagyp@med.unideb.hu; 3 MTA-DE Cell Biology and Signaling Research Group, Faculty of Medicine, University of Debrecen, 4032 Debrecen, Hungary

**Keywords:** polyphenols, breast cancer, prevention, apoptosis, cell cycle, signaling pathways

## Abstract

The review will discuss in detail the effects of polyphenols on breast cancer, including both the advantages and disadvantages of the applications of these natural compounds. First, we focus on the characterization of the main classes of polyphenols and then on *in vitro* and *in vivo* experiments carried out in breast cancer models. Since the therapeutic effects of the administration of a single type of polyphenol might be limited because of the reduced bioavailability of these drugs, investigations on combination of several polyphenols or polyphenols with conventional therapy will also be discussed. In addition, we present recent data focusing on clinical trials with polyphenols and new approaches with nanoparticles in breast cancer. Besides the clinical and translational findings this review systematically summarizes our current knowledge about the molecular mechanisms of anti-cancer effects of polyphenols, which are related to apoptosis, cell cycle regulation, plasma membrane receptors, signaling pathways and epigenetic mechanisms. At the same time the effects of polyphenols on primary tumor, metastasis and angiogenesis in breast cancer are discussed. The increasing enthusiasm regarding the combination of polyphenols and conventional therapy in breast cancer might lead to additional efforts to motivate further research in this field.

## 1. Introduction

Carcinogenesis is a complex and multistage process which, for easier comprehension, might be classified to the following three main steps: initiation, promotion and progression [1,2]. *Tumor initiation* was considered a rapid and irreversible process correlated with the exposure to the carcinogenic agent, distribution of the carcinogenic agent to the cells, the interaction of the carcinogen or its metabolites with DNA, leading in the end, to the appearance of the genotoxic effects. The second step, *cancer promotion*, has been associated with proliferation of pre-neoplastic cells and it is considered a prolonged and possibly reversible stage. The third step, *tumor progression* or neoplastic transformation is a process accompanied by tumor growth, invasiveness and metastasis [1,2]. In accordance with the above mentioned stages of carcinogenesis, a chemopreventive agent will be able to inhibit, delay or reverse tumorigenesis or pre-malignant lesions [3,4]. De Flora and collaborators divided cancer chemopreventive agents to three classes: primary, secondary and tertiary. *Primary prevention* blocks the occurrence of the disease in healthy individuals by inhibiting mutagenesis and cancer initiation as well as tumor promotion. *Secondary prevention* acts during preclinical or early stages of tumorigenesis by the inhibition of tumor progression (*i.e.*, antioxidant activity, modulation of signal transduction, modulation of hormones and immune status, inhibition of angiogenesis). *Tertiary prevention* is achieved by the inhibition of invasion and metastasis in cancer patients after therapy and it includes the modulation of cell-adhesion molecules, the inhibition of proteases involved in extracellular matrix degradation and the activation of anti-metastatic genes [5,6]. Therapeutic effects in allopathic, mainstream medicine are often achieved by acting on a single target. To the contrary, the effects of dietary agents must be seen as a set of several effects rather than a single biological response and for this reason they might act on the entire process of malignant transformation [7]. Since several authors have hypothesized that “multiple weak hits confuse the complex system” [8,9], the pleiotropic effect of the polyphenols had been considered appropriate to delay and to fight the carcinogenic processes in the breast tissue [10,11].

## 2. Breast Cancer—General Aspects

Despite the progress in the fight against malignancy, breast cancer incidence has still increased worldwide, with more than 1.3 million cases associated with 450,000 deaths per year [12]. Apocrine glands located in the skin were identified as the evolutionary origins of the mammary glands and their main function is to provide nutrients to the newborn [13,14]. Since factors involved in the development of the mammary gland are very similar to those required for a malignant process, a better understanding of the normal physiology of breast development might help in deciphering the biology of tumorigenesis [15]. Organogenesis of the mammary gland starts in the embryonic life followed by a period of inhibition till puberty, when the mammary ducts are elongated and branched due to the presence of invading structures called terminal end buds (TEB); the most intense morphological and physiological changes take place during the adult life, due to pregnancy, lactation and after lactation period when 80%–90% of the epithelial cells might be eliminated through an apoptotic process in a few days [13,15,16].

In breast cancer several clinical features, such as age, tumor size, axillary lymph node status, hormone and human epidermal growth factor receptor 2 (HER2) receptor status, histological grade or the presence of metastasis are routinely investigated in order to provide the patients with the best treatment [17]. A major challenge in the treatment of breast cancer is its high heterogeneity from patient to patient which initiated its classification into three major molecular subtypes, according to estrogen receptors (ER), progesterone receptors (PR) and HER2: hormone receptor positive with luminal A (ER+PR+HER2−) and luminal B (ER+PR+HER2+) phenotypes, HER2 positive (ER−PR−HER2+) and triple negative/basal-like (ER−PR−HER2−) [17,18,19]. The biomarker profile may be more complex by including additional molecules to the previous classification: nuclear protein necessary for cell proliferation (Ki67), cytokeratin 5/6 (CK5/6) and epidermal growth factor receptor (EGFR) [17,18]. About 70% of breast cancers are estrogen receptor positive [20]. In addition to the application of conventional therapy, the non-specific chemotherapy of breast cancer is supplemented with targeted drugs according to the molecular subtypes of the disease, namely: (i) the use of estrogen antagonists, like tamoxifen, fulvestran or aromatase inhibitors in estrogen positive tumors and (ii) the administration of anti-HER2 antibodies, like trastuzumab and tyrosine kinase inhibitors (TKI), like lapatinib in HER2 positive tumors. Unfortunately, the application of systemic, conventional chemotherapy: anthracycline family (doxorubicine) and taxane family (paclitaxel) remains the only option in the treatment of triple negative breast cancer (TNBC) [21,22]. In addition to the therapy targeted at the cancer cells themselves an antibody against vascular endothelial growth factor receptor (VEGFR), bevacizumab, which inhibits angiogenesis, may also be administrated in combination with the above mentioned drugs [21]. However, 25%–45% of patients develop metastatic disease [22,23]. Besides the beneficial effects of anti-cancer drugs, their side effects are significant; for instance, administration of doxorubicine or taxol induces cardiotoxicity [24,25,26] and combination of anti-cancer drugs can result in massive adverse effects [27]. Similarly, longtime administration of anti-cancer drugs leads to drug resistance and further the development of recurrences [27]. The use of natural compounds from edible fruits or vegetables presents an alternative way since they are pleiotropic molecules with fewer side effects than conventional therapy [27].

## 3. Polyphenols—General Aspects

Plants produce primary and secondary metabolites with the first category involved in essential functions such as: photosynthesis, respiration and development, while the second one being responsible for attracting pollinators, protecting against ultraviolet radiation and for defense against herbivores and pathogens. Additionally, secondary metabolites provide humankind with different drugs, antibiotics and herbicides [28,29]. The prolonged intake of secondary plant metabolites has demonstrated favorable impact on cancer, cardiovascular diseases, type II diabetes or neurodegenerative diseases [29,30,31,32]. Polyphenols are secondary plant metabolites found in fruits, vegetables, spices, nuts, grains, tea, coffee or wine and they are recognized for their powerful antioxidant properties [28,30]. Nevertheless, the pro-oxidant effect of polyphenols was also observed, since after donating an electron or a hydrogen atom, they become reactive species capable of interacting with other molecules [33,34,35]. Polyphenols are chemical compounds with more than one hydroxyl functional group (–OH) attached to an aromatic ring [36]. More than 8000 species of polyphenols have been identified in the plant kingdom and they are regularly present as glycosylated forms with one or more sugar residues conjugated to a hydroxyl group or the aromatic ring [37,38].

Polyphenols are classified based on several criteria in line with their source, biological function or chemical structure. According to the most recent classification polyphenols can be divided into two major groups: (1) flavonoids; and (2) non-flavonoids [28,39]. Flavonoids include different subclasses and the most representative ones are flavonols, flavones, flavan-3-ols, anthocyanidins, flavanones and isoflavones [38]. Non-flavonoids comprise the following main classes: phenolic acids (benzoic acids and cinnamic acids), stilbenes, lignans, tannins and other polyphenols (including curcumin, rosmarinic acid, gingerol, *etc.*) [28,31,36]. Out of all polyphenol classes, 60% are represented by flavonoids and 30% by phenolic acids [31,40].

*Flavonoids* are the most representative group of polyphenols. They consist of 15 carbon atoms (C_6_–C_3_–C_6_) characterized by two benzene rings joined by a three carbon chain forming an oxygenated heterocycle [28,29,38]. The main dietary sources of flavonoids are fruits, vegetables, medicinal herbs, spices, tea, coffee and wine. The daily intake of flavonoids is variable according to each subclass, namely: 0.1–1.2 mg (isoflavones), 0.3–1.6 mg (flavones), 5.4–27.4 mg (flavonols), 20.4–50.6 mg (flavanones), 12–189.2 mg (flavan-3-ols) and 180–215 mg (anthocyanins) [28]. The most well-known flavonoid sub-classes are presented in Table 1 including members, chemical structure and dietary sources. 

*Non-flavonoids.* The main non-flavonoid phenolic classes (Table 2) are represented by: phenolic acids (hydroxybenzoates and hydroxycinnamates); polyphenolic stilbenes; polyphenolic tannis with gallic acid as precursor of hydrolysable tannins (gallotannins, ellagitannis); other polyphenols: curcumin, rosmarinic acid, gingerol *etc.* [39].

## 4. *In Vitro* Effect of Polyphenols

### 4.1. Antioxidant Activity of Polyphenols

Due to their ability to display both anti-oxidant and pro-oxidant activity polyphenols have been considered “double-edge swords” [41]. The antioxidant activities of polyphenols have been broadly studied, but they still need to be better understood. The main mechanisms of the antioxidant activity of polyphenols can be summarized as follows: (i) free radical scavenger; (ii) metal chelating abilities; (iii) inhibition of several types of oxidases (lypooxygenase, cyclooxygenase *etc.*); and (iv) stimulation of enzymes with anti-oxidant properties (superoxide dismutase, catalase *etc.*) [42]. The free radical scavenger activity of polyphenols was strongly correlated with their chemical structure, namely the presence of: (i) catechol (1,2-dihydroxibenzen) group on the B-ring; (ii) 2,3-double bond conjugated with the 4-oxo-function (oxygen atom double bonded to carbon) of the carbonyl group in the C-ring; and (iii) hydroxyl groups at positions 3 and 5 [41]. On the other hand, the main pro-oxidant mechanisms of polyphenols have been associated with: (i) generation of reactive oxygen species which in turn will induce mitochondrial dysfunction in connection with apoptotic cell death [43]; and (ii) oxidative DNA damage [44].

**Table 1:** Main classes of flavonoids with chemical structure, representative members and dietary sources [36,39,45,46].

Class of Flavonoid	Chemical Structure	Representative Members	Dietary Sources
**Flavonoids C_6_–C_3_–C_6_**	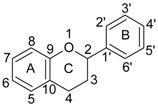		
Flavonols	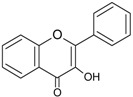	Quercetin Kaempferol Myricetin	Onions (*Allium cepa*), spinach (*Spinacia oleracea*), cauliflower (*Brassica oleracea* Botrytis Group), strawberries (*Fragaria ananassa*)
Flavones	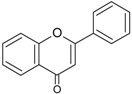	Apigenin Luteolin	Celery (*Apium graveolens*), parsley (*Petroselinum crispum*), artichoke (*Cynara scolymus*)
Flavan-3-ols/proto-anthocyanidins	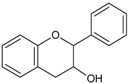	Epicatechin Epigallocatechin Epigallocatechin-3-*O*-gallate (EGCG) Procyanidin 2	Apricots (*Prunus armeniaca*), sour cherries (*Prunus cerasus*), grapes (*Vitis* ssp.), blackberries (*Rubus* ssp.), apples (*Malus domestica*) dark chocolate—seeds of cocoa (*Theobroma cacao*), mint (*Mentha rodundifolia*), basil (*Ocimum basilicum*), rosemary (*Rosemarinus officinalis*), sage (*Salvia officinalis*), dill (*Anetheum graveolens*), green tea (*Camellia sinensis*), hazelnuts (*Corylus avellana*), pecans (*Carya illinoensis*), almonds (*Prunus dulcis*), pistachios (*Pistachio vera*), walnuts (*Juglans* ssp.)
Anthocyanidins/Anthocynins	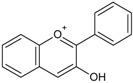	Cyanidin Pelargonidin Delphinidin Malvidin	Red grapes (*Vitis labrusca, Vitis vinifera*), cranberry (*Vaccinium macrocarpon*), blackberry (*Rubus* ssp.), elderberry (*Sambucus nigra*), blueberry (*Vacciunium corymbosum*), blackcurrant (*Ribes nigrum*), sweet cherries (*Prunus avium*), sour cherries (*Prunus cerasus*), plums (*Prunus domestica*), peaches (*Prunus persica*)
Flavanones	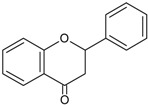	Naringerin Hesperitin	Orange (*Citrus sinensis*), lemon (*Citrus lemon*), mandarin (*Citrus reticulate*), grapefruit (*Citrus paradisi*), tomatoes (*Solamun lycopersicum*)
Isoflavones	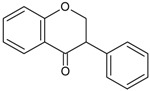	Genistein Daidzein Glycitin	Soybean (*Glycine max*), beans (*Phaseolus vulgaris*), green peas (*Pisum sativum*)

**Table 2:** The main classes of non-flavonoid polyphenols with chemical structure, representative members and dietary sources [36,39,45].

Class of Non-Flavonoids	Chemical Structure	Representative Members	Dietary Sources
Phenolic acids—Benzoic acids/hydroxybenzoates C_6_–C_1_	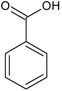	Gallic acid *p*-hydroxy-benzoic Syringic acid Vanillic acid	Clove buds (*Eugenia caryophyllata*) Grains: wheat (*Triticum vulgare*), rice (*Oryza sativa*), oat (*Avena sativa*), rye (*Secale cereale*), barley (*Hordeum vulgare*) Dates (*Phoenix dactylifera*)
Phenolic acids—Cinnamic acids/hydroxycinnamates C_6_–C_3_	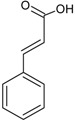	*p*-coumaric acid Caffeic acid Ferulic acid Chlorogenic acid	Apples (*Malus domestica*) Dates (*Phoenix dactylifera*) Green coffee beans (*Coffea arabica*) Carrots (*Daucus carota*)
Stilbenes C_6_–C_2_–C_6_	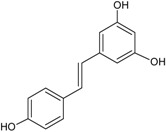	Resveratrol	Red wine, peanuts (*Arachis hypogaea*), red cabbage (*Brassica olearaceae* Capitata Group), spinach (*Spinacia oleracea*)
Other polyphenols	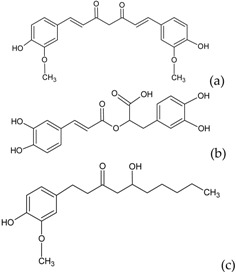	Curcumin (a) Rosmarinic acid (b) Gingerol (c)	Turmeric (*Curcuma longa*) Rosemary (*Rosmarinus officinalis*) Ginger (*Zingiber officinale*)

Administration of EGCG in reduced doses (10, 20 µM) was associated with decreased levels of reactive oxygen species (ROS) in MCF-10A transformed with a combination of carcinogens [47] and in Hs578T breast cancer cells, while higher doses (100 µM) increased the production of ROS in Hs578T breast cancer cells [48]. On the other hand, 10 µM EGCG failed to modulate the level of superoxide dismutase or catalase, but increased the expression of quinone reductase 1 in MCF-7 breast cancer cells. At the same time, resveratrol displayed similar effects to EGCG on superoxide dismutase, catalase and quinone reductase 1 in MCF-7 cell line, but its dose dependence was opposite to that of EGCG [49]. Incubation of MCF-7 cells with 10 µM resveratrol induced an increase in ROS production, while 100 µM resveratrol decreased it [50]. Administration of low doses of quercetin (0.62 µM for 24 h) did not change the levels of ROS in MCF-7 and MDA-MB-231 breast cancer cells [51], but higher doses of quercetin increased the level of ROS in MDA-MB-468 breast cancer cell line [52]. Physiological doses of genistein (1 µM) administrated for 48 h increased copper and zinc superoxide dismutase (CuZn-SOD) and manganese superoxide dismutase (Mn-SOD) in the T47D cell line, but not in MCF-7 cells and it did not significantly influence catalase levels suggesting that the anti-oxidant effect of genistein was cell line dependent which may be correlated with the ERα/ERβ ratio [53]. The apoptotic activity of genistein in breast cancer cells was correlated with a ROS production. Thus, administration of 50 µM genistein led to apoptosis, while ROS scavengers induced the opposite effects in MDA-MB-231, MDA-MB-468, but not in MCF-10A cells [54]. Treatment with 10–100 µM curcumin induced time-dependent increase in ROS production in MCF-7 and MDA-MB-231 breast cancer cell lines, demonstrating a pro-oxidant effect [50]. On the contrary, curcumin prevented the increased production of ROS induced by nickel oxide nanoparticles in MCF-7 cells [55]. The contradiction regarding the pro- or antioxidant effects of polyphenols may be the result of differences in the cell models or experimental conditions. However, some of the polyphenols (EGCG) displayed double effects as hypothesized, both anti- and pro-oxidant, and these effects were correlated with dose-dependent administration. Additional data in breast cancer cell lines and animal models are required to clarify and confirm the previous results.

### 4.2. Polyphenols and Aromatase Inhibitor Activity

**Figure 1:**
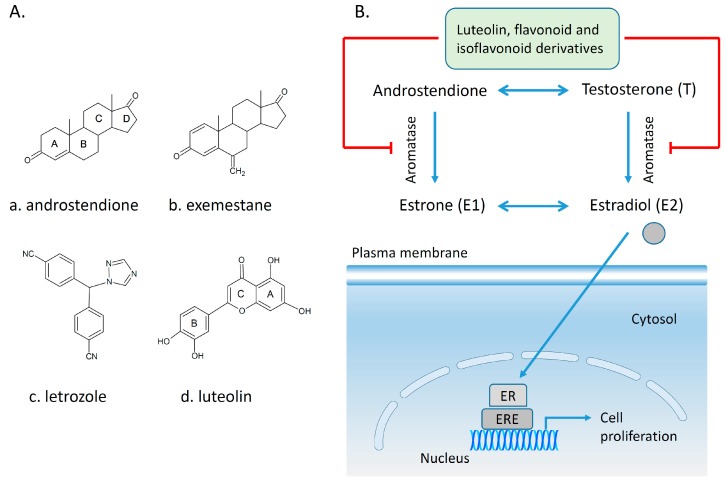
Poyphenols as aromatase inhibitory molecules. (**A**) Chemical structure of aromatase inhibitors and natural substrates. a. androstenedione (substrate for aromatase); b. exemestane (3rd generation steroidal inhibitor); c. letrozole (3rd generation non-steroidal inhibitor); d. luteolin (flavonoid) [56,57]; (**B**) Schematic mechanism of activity of aromatase inhibitors in relation to nuclear estrogen receptors. Androgens (androstendione and testosterone) are converted to estrogens (estrone and estradiol) by aromatase; estradiol enters the nucleus and binds to estrogen receptors (ER) which interacts with estrogen response elements (ERE) triggering cell proliferation. So far, luteolin, flavonoid and isoflavonoid derivatives have been found to inhibit aromatase activity in breast cancer cells [64,66,67].

The cytochrome P450 enzyme complex or aromatase is able to convert androgens (C19) to estrogens (C18) [56,57]. Under normal conditions, estrogens are responsible for reproduction, neuroendocrine activity and for the development of female reproductive organs [58,59]. While aromatase is physiologically expressed in the ovaries of premenopausal women, in the placenta of pregnant women, in the adipose tissue of postmenopausal women and in breast tissue, it is pathologically produced by cancer-associated fibroblasts in breast cancer. Several factors are responsible for the activation of aromatase in breast tissue: prostaglandin-E2 (PGE2) released by breast cancer cells or inflammatory cells and cytokines (IL-6, IL-11 and TNFα) produced by inflammatory cells [56]. Since 70% of breast cancers are estrogen dependent, *aromatase inhibitors* are used for treating these tumors. Aromatase inhibitors can be classified into two groups (Figure 1A): (i) *steroidal aromatase inhibitors*, whose structure is similar to that of the natural substrate, adrostendione. They act as false substrates and are processed to an intermediate binding irreversibility to the active site. The mechanism of action is referred to as “suicide inhibition”. Formestane (2nd generation) and exemestane (3rd generation) are examples of such inhibitors; (ii) *non-steroidal aromatase inhibitors*, which are reversible, competitive inhibitors since they bind to the iron atom in the heme group of aromatase with their nitrogen atom present in imidazole, triazole, pyrimidine or pyridine groups. Examples of non-steroidal inhibitors include: aminoglutethimide (1st generation), fadrozole (2nd generation), triazoles (3rd generation). The third generation inhibitors: anastrozole and letrozole are 100–3000 times more active than aminoglutethimide [56,57]. Besides the main steroidal and non-steroidal aromatase inhibitors, flavonoids and flavonoid derivatives attracted attention due to their ability to inhibit aromatase activity [60,61]. Flavonoids and flavonoid derivatives contain two structural features which can contribute to their aromatase inhibitory activity: (i) A and C rings of the flavonoids may mimic D and C rings of the aromatase substrate (androstendione) and (ii) oxo-group in C4 position was considered essential to bind the iron atom of the heme group of aromatase [57]. At the same time, it has been noticed that flavones and flavanones display higher inhibitory activity compared to isoflavones and isoflavanones [62]. Nevertheless, a major drawback of flavonoids and flavonoid derivatives is their pleiotropic effect. Their multiple interactions with many biological molecules set limitations for their therapeutic applications [63].

A recent report concluded that luteolin, a dietary flavonoid, demonstrated aromatase inhibitor activity at low concentrations (2.44 µM) [64]. However, another dietary flavonoid, hesperitin increased the expression of aromatase at the mRNA level suggesting that dietary flavonoids could regulate aromatase expression differentially [65]. The results of a high-throughput study screening of 7000 compounds identified an imidazolyl quinoline derivative of flavonoids with aromatase inhibitor effect at a concentration of 0.81 µM decreasing the proliferation of T47D breast cancer cells [66]. Based on the structure of isoflavanones a new class of aromatase inhibitors has been developed with the ability to inhibit aromatase activity in the concentration range of 0.26 to 5.8 µM [67]. Polyphenols from black tea have been shown to inhibit aromatase activity and to decrease the proliferation of dehydroepiandrosterone-induced MCF-7 cells by one study only [68]. Satoh *et al.* also concluded that several components of green tea extract, including EGCG have aromatase inhibitory activity with IC_50_ values in the micromolar range [69]. A schematic mechanism of action in case of polyphenols as inhibitors of aromatase is shown in Figure 1B.

Beside these promising results, genistein in an *in vitro* model based on co-culture between MCF-7 cells and primary breast adipose fibroblasts increased aromatase activity and canceled the aromatase inhibitor activity of fadrozole [70]. So far the efficacy of flavonoids and their derivatives has not been conclusively shown, due to the lack of data or contradictory results. Additional experiments including *in vitro* and *in vivo* assays as well as clinical trials are still required.

### 4.3. Reversal of Glycolytic Metabolism by Polyphenols

Since 1924 it has been established that cancer cells display intense glycolysis even in the presence of oxygen and the effect was coined, according to its discoverer, Warburg effect. This effect can be observed nowadays during positron emission tomography with [^18^F]-fluoro-2-deoxyglucose (^18^F-FDG), since cancer cells have a more rapid uptake of glucose compared to the normal ones [71]. In order to obtain energy, cancer cells prefer *aerobic glycolysis* (*i.e.*, glycolysis even in the presence of oxygen), while normal cells favor *mitochondrial oxidative phosphorylation*. As a result cancer cells obtain only two molecules ATP per molecule glucose while normal cells may acquire 32 molecules of ATP per molecule glucose [72,73]. The obviously inefficient production of ATP raises the question: why cancer cells prefer a low efficiency of ATP generation although they are highly proliferative systems? A possible answer may be related to the fact that during aerobic glycolysis ATP is generated with low efficiency, but at higher rate; on the contrary in the mitochondrial oxidative phosphorylation ATP is generated with high efficiency, but at slower rate. In addition, cancer cells also exploit the glycolytic pathway to obtain precursors for nucleotides, fatty acids and amino acids which are required for nucleic acid generation, membrane biogenesis and protein synthesis in highly proliferating systems [74,75].

**Figure 2:**
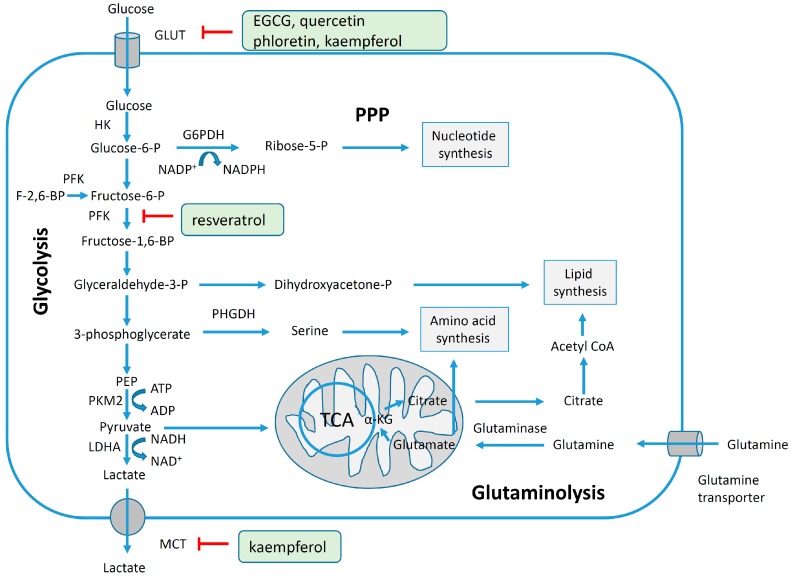
Schematic representation of glycolysis, the pentose phosphate pathway (PPP), glutaminolysis and lipid synthesis and their inhibition by polyphenols in breast cancer cells. During glycolysis, glucose enters the cell through the glucose transporter (GLUT) and then it is oxidized to pyruvate followed by reduction to lactate. Lactate is released from the cell through the monocarboxylate transporter (MCT). The rate of aerobic glycolysis in cancer cells is increased in detriment of ATP production. The intermediate compounds from the glycolytic pathway are further used to synthetize nucleotides, amino acids and lipids required for the proliferation of cancer cells [72,75,76,80]. The glucose transporter can be inhibited by EGCG, quercetin, phloretin, kaempferol [81,82,83], PFK by resveratrol [84], and MCT by kaempferol [83]. Abbreviations: PEP, phosphoenolyruvate; TCA, tricarboxylic acid cycle; F-2,6-BP, fructose-2,6-biphosphate; α-KG, alpha-ketoglutarate; HK, hexokinase; G6PDH, glucose-6-phosphate dehydrogenase; PFK, phosphofructokinase; PHGDH, 3-phosphoglycerate dehydrogenase; PKM2, pyruvate kinase isoform M2; LDHA, lactate dehydrogenase A; Acetyl CoA, acetyl coenzyme A; ADP, acid adenosine diphosphate; ATP, acid adenosine triphosphate; NADP+, nicotinamide adenine dinucleotide phosphate (oxidized); NADPH, reduced nicotinamide adenine dinucleotide phosphate; NADH, reduced nicotinamide adenine dinucleotide; NAD+, nicotinamide adenine dinucleotide (oxidized).

Figure 2 shows schematically the main pathways preferentially utilized by cancer cells: aerobic glycolysis, fatty acid synthesis and glutaminolysis. Briefly, glucose after the cellular uptake is phosphorylated to glucose-6-phosphate by hexokinases; glucose-6-phosphate during the pentose phosphate pathway can be metabolized to ribose-5-phosphate in the presence of glucose-6-phosphate dehydrogenase and further used for nucleotide synthesis. In a series of consecutive steps glucose-6-phosphate is metabolized to phosphoenolpyruvate through the intermediate products fructose-6-phosphate, fructose-1,6-biphosphate, glyceradehyde-3-phosphate and 3-phosphoglycerate. In the presence of the M1 (adult) splice variant of pyruvate kinase phosphoenolpyruvate is transformed to pyruvate and further on pyruvate in the presence of lactate dehydrogenase A is metabolized to lactate which will be released by the cells through the monocarboxylate transporters [72,75,76]. On the other hand, tumor cells express the M2 (embryonic) splice variant of pyruvate kinase which performs the conversion of phophoenolpyruvate to pyruvate much more slowly resulting in the accumulation of all glycolytic intermediates before pyruvate [77]. Inhibition of the expression of the M2 isoform of pyruvate kinase not only resulted in reversal of the Warburg effect, but also led to a reduced ability to form tumors in nude mice [78].

Intermediary molecules of this pathway can be further utilized in anabolic processes, e.g., glyceraldehyde-3-phosphate for lipid synthesis, 3-phosphoglycerare for amino acid synthesis and pyruvate for fatty acid synthesis via the tricarboxylic cycle. In glutaminolysis glutamine is converted to glutamate by glutaminase and then to α-ketoglutarate. Cancer cells prefer glutamine as the main source of carbon in the tricarboxylic cycle [75]. Since cancer cells are dependent on aerobic glycolysis [78], the enzymes contributing to glucose metabolism may represent an attractive target for cancer therapy. Nevertheless an open question remains here: is it appropriate to target molecules expressed both in normal and in cancer cells non-selectively? So far, only a few studies have described the ability of polyphenols to inhibit the glycolytic pathway in breast cancer cells. 150 µM resveratrol administrated for 24 h decreased ^18^F-FDG uptake by 35% in T47D mammary cancer cells, but the mechanism of action remains to be clarified [79]. The enzyme 6-phosphofructo-1-kinase (PFK) may be considered one of the main enzymes in the glycolytic pathways, since its levels have been linked to glucose intake. The administration of resveratrol (1–100 µM for 24 h) to MCF-7 breast cancer cells was associated with decreased glucose uptake, increased lactate production, decreased intracellular ATP content and inhibition of PFK activity. Since cancer cells express all isoforms of PFK the mechanism of action of resveratrol must be characterized in terms of its effect on different isoforms.

Administration of resveratrol induced the dissociation of the tetrameric PFK-M isoform, isolated from rabbit skeletal muscle to a less active form, the dimer [84]. Glucose uptake was investigated in MCF-7 and MDA-MB-231 breast cancer cells using ^3^H-2-deoxy-d-glucose (^3^H-DG). The consumption of ^3^H-DG in breast cancer cells was found to be suppressed by cytochalasin B, a glucose transporter inhibitor, moderately stimulated by insulin and independent of sodium suggesting that it was mainly mediated by the GLUT family of transporters rather than by sodium-glucose transporter. Administration of EGCG and quercetin suppressed ^3^H-DG uptake in a concentration-dependent manner in both cells lines and inhibited lactate production only in MCF-7 cells, suggesting that EGCG and quercetin might be considered possible therapeutic/adjuvant agents in breast tumors [81]. Recent data demonstrated that two flavonoids, quercetin and phloretin (0.6–300 µM) inhibited glycolysis indicated by increased glucose and decreased lactate concentrations in the cell culture media of MCF-7 and HBL100 breast cancer cells after 24 h of exposure [82]. The effect of several polyphenols (myricetin, chrysin, genistein, resveratrol and kaempferol with concentration range 10–100 µM) was investigated in a short-term experiment (26 min), resulting in the selection of kaempferol as the most potent inhibitor of ^3^H-DG uptake in MCF-7 cells (IC50 4 µM). In the long-term experiment (24 h) 30 µM kaempferol inhibited ^3^H-DG uptake and decreased GLUT1 mRNA levels by 40% associated with inhibition of monocarboxylated transporter (MCT-1) [83]. Taken together the above mentioned reports suggest that polyphenols may be considered as natural inhibitors of the glycolytic pathway in cancer cells (Figure 2).

### 4.4. Regulation of Cell Cycle and Apoptosis by Polyphenols

**Figure 3:**
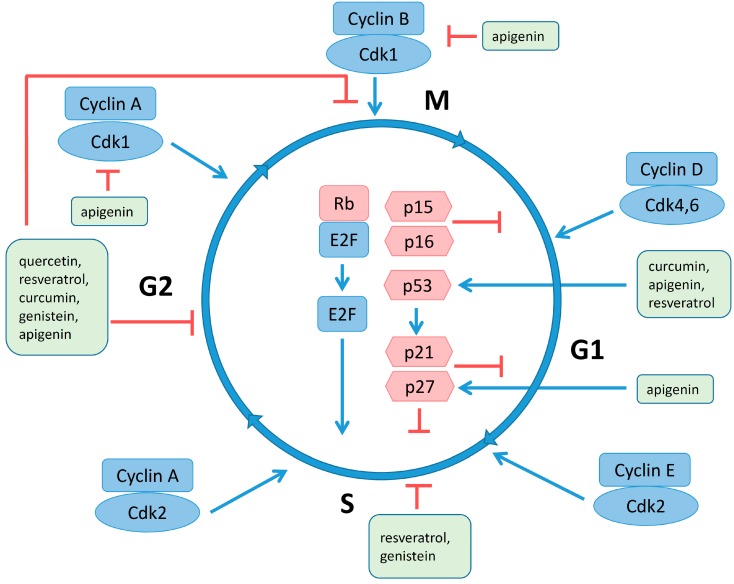
The effect of polyphenols on cell cycle. Cell cycle phases with (i) stimulatory proteins: cyclin-Cdk complexes and eukaryote transcription factor (E2F) in blue and (ii) inhibitory proteins: retinoblastoma protein (Rb), p15, p16, p21, p27 and p53 in red; phosphorylation of Rb will release E2F which further stimulates cell cycle progression; p53, the tumor suppressor protein is responsible for stimulation of p21 and p27 expression, two inhibitory proteins of the cyclin-Cdk complexes [103,104,105,106,107,108]. Quercetin, resveratrol, curcumin, genisteins and apigenin arrest the cells in G2/M phase, while resveratrol and genistein in S phase; apigenin inhibits Cyclin A, B-Cdk1 complexes, and stimulate p21 and p27; curcumin, apigenin and resveratrol increase the activity of p53 [100,101,102,109,110,111,112,113]. Blue and red lines indicate stimulation and inhibition, respectively, of the process (for the sake of simplicity only the major proteins involved in cell cycle regulation are shown).

**Figure 4:**
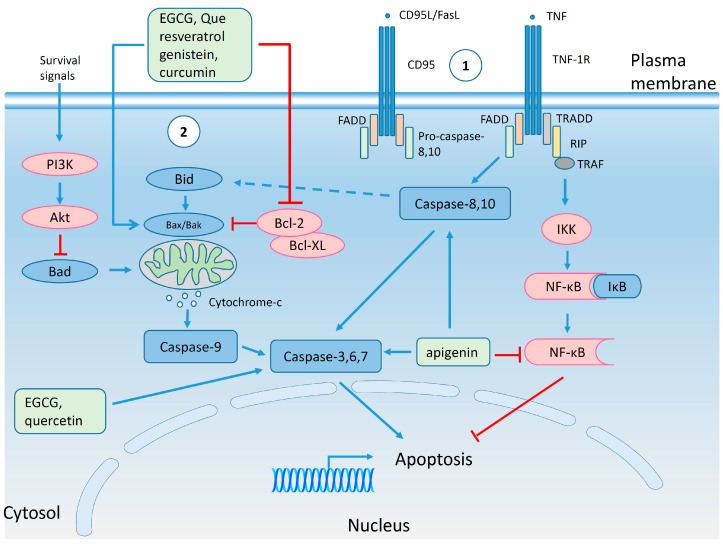
The effect of polyphenols on apoptotic signaling pathways. Extrinsic (1) and intrinsic (2) apoptosis pathways are shown in the figure. The proteins with pro-apoptotic activity (caspases, Bax, Bak, Bid, Bad, IkB) are colored in blue, while the proteins with anti-apoptotic action (Bcl-2, Bcl-XL, PI3K, Akt, IKK, NF-kB) in red. EGCG, resveratrol, genistein and curcumin can inhibit anti-apoptotic proteins (Bcl-2) and stimulate the pro-apoptotic ones (Bax, Bak); caspases are stimulated by EGCG, quercetin and apigenin; the anti-apoptotic activity of NF-kB can be inhibited by apigenin [115,116,117,118,119,120,121]. Blue and red lines indicate stimulation and inhibition, respectively, of the process (for the sake of simplicity only the major proteins of the apoptosis pathways are shown).

The cell cycle (Figure 3) is a regulated sequence of events in which the mother cell divides generating two daughter cells. During all these events pro- and anti-proliferative factors compete with each other determining whether a cell divides, stops in the cell cycle or dies. Factors favoring proliferation may be of intracellular (e.g., complexes between cyclins and cyclin dependent kinases (Cdk), regulatory proteins, checkpoints *etc.*) or extracellular origin (e.g., growth factors and cell adhesion molecules) [85,86]. Beside stimulatory molecules (cyclin-Cdk complexes and eukaryote transcription factor, E2F), the cell cycle is regulated by Cdk inhibitory proteins (retinoblastoma tumor suppressor protein (Rb), p15, p16, p21, p27 and p53) [87,88]. In tumor cells the cyclin-Cdk complexes are overexpressed, while the inhibitory proteins display a low expression [89]. Although cells attempt to correct mutations in DNA, extensive damage revealed at cell cycle check points may lead to programmed cell death, *i.e.*, apoptosis [90,91]. Apoptosis (Figure 4) is initiated by two interconnected signaling routes: (i) extrinsic pathway which operates through the cell surface death receptors; and (ii) intrinsic pathway which involves the disruption of mitochondrial membrane integrity, both of them having as common effectors a family of cysteine aspartic proteases (caspases) [92,93]. The extrinsic pathway involves the engagement of death receptors. Two main types of death receptor complexes are presented in Figure 4: (i) first group, death-inducing signaling complexes (DISC) includes cluster of differentiation (CD) molecules, like CD95 or tumor necrosis factor receptor superfamily member 6 (Fas) with its ligand (CD95L/FasL), Fas-associated death domain protein (FADD) and pro-caspase-8,10; (ii) the second group, represented by tumor necrosis factor receptor 1 (TNFR1), which after the binding of tumor necrosis factor (TNF) will recruit receptor interacting protein (RIP), TNFR1-associated death domain protein (TRADD) and TNFR-associated factor (TRAF) [94,95]. Death receptors, through activated caspase-8,10, will further trigger the executioner caspases-3,6,7 which will lead to the formation of apoptotic bodies [96,97]. However, TNFR1 in connection with the nuclear factor kappa-light-chain-enhancer of activated B cells (NF-κB) pathway can prevent TNF induced apoptosis [98]. The intrinsic pathway acts through B-cell lymphoma 2 protein (Bcl-2) family of proteins and can be triggered by diverse stress factors which will activate Bcl-2 homology domain 3-interacting domain death agonist (Bid), which, in turn, activates Bcl-2 associated X protein (Bax)/Bcl-2 homologus killer (Bak) oligomeric complexes localized in the mitochondrial membrane. These complexes are also responsible for the efflux of cytochrome *c* from mitochondria. Cytochrome *c* together with apoptotic protease activator factor 1 (APAF-1) will form the apoptosomes, which will activate caspase-9 connected further with executioner caspases-3,6,7. Apoptotic signals can be inhibited by anti-apoptotic proteins: Bcl-2 and B-cell lymphoma-extra-large protein (Bcl-XL). Sometimes there is cross-talk between the extrinsic and intrinsic pathways, through caspase-8 and Bid. Activation of the survival pathway by phosphatidylinositol 3-kinase (PI3K)/protein kinase B (Akt) can inhibit the pro-apoptotic protein, Bcl-2-associated death promoter (Bad) [95].

In the MCF-7 breast cancer cell line the cell cycle progression may be stimulated by the cooperation between 17β-estradiol (E2) and insulin-like growth factor-I (IGF-I). Thus, E2 and IGF-1 can stimulate the expression of cyclin D1, Cdk2, Cdk4 [99]. Polyphenols were shown to inhibit proliferation and cell cycle progression. Administration of 5 and 10 µM quercetin-3-methyl ether to wild-type and lapatinib-resistant SK-BR-3 breast cancer cells for 16 and 48 h induced significant accumulation of cells in the G2/M phase, which was correlated with increased levels of cyclin B1/p-cyclin B1 (Ser 147), cell division cycle 225 proteins (Cdc225): Cdc225/p-Cdc225 (Ser216) and check point kinase (Chk) Chk1/p-Chk1 (Ser345) in sensitive SK-BR-3 cells, but not in resistant ones [100]. In MDA-MD-453 breast cancer cell line 100 µM quercetin applied for 24 h induced an increase in the fraction of cells in the G2/M phase associated with a decrease in the fraction of cells residing in the G1 phase [101]. Administration of 25 and 50 µM resveratrol for 3 days induced the increase of the percentage of MCF-7 cells in the G2/M phase, while similar concentrations lead to an accumulation of cells in the S phase in correlation with a reduction in the fraction of cells in the G1 phase in MDA-MB-231 breast cancer cells, suggesting cell line dependent effects [102].

Curcumin induced a series of effects related to the inhibition of the mitotic spindle in MCF-7 breast cancer cell line including depolymerization of mitotic microtubules, modification of microtubule kinetochore attachment, disturbed mitotic spindle structure and perturbed the localization of a member of the kinesin-5 subclass of kinesins (Eg5). Moreover, curcumin induced monopolar spindle formation associated with the accumulation of mitotic arrest deficient 2 proteins (Mad2) which in turn activated the mitotic checkpoints. Also, administration of 35 µM curcumin for 4 and 8 h induced mitotic arrest of MCF-7 cells [109]. Due to increased structural similarity between E2 and genistein, the polyphenol greatly influenced the cell cycle progression. In a more complex experiment Tominaga and coworkers also investigated the effect of genistein on the cell cycle. The experiments were started with four mouse cell lines derived from mammary tumors and the administration of 20–80 µM genistein inhibited the growth of all the mammary cancer cells investigated after 3 days of exposure, while long-term exposure (7 days) to 15 µM genistein significantly decreased the survival of the W780 cell line only. In accordance with previous data, administration of genistein for 3 days increased the number of cells in the S/G2 phase accompanied by a reduction in the fraction of cells in G1. Besides the blocking in cell cycle progression, DNA damage was also increased suggested by the increased level of histone 2A variant phosphorylated at serine 139 (γ-H2AX). This phenomenon may have led to the appearance of cells in the sub-G1 peak. The increased polyploidy (4*n* and 8*n*), observed also after genistein treatment, may have been related to abnormal chromosomal structures in anaphase and the inhibition of topoisomerase II, an enzyme responsible for the segregation of chromosomes [110]. The effect of genistein was recently investigated on human breast cancer cell lines, MCF-7 (high ERα/ERβ ratio), T47D (low ERα/ERβ ratio) and MDA-MB-231 (ER-negative) indicating that the treatment with genistein at physiological concentrations (1 µM) for 48 h increased the fraction of cells in the S/G2/M phases at the expense of the G1 phase in MCF-7 cells with no effects observed in T47D and MDA-MB-231 cells suggesting that a high ERα/ERβ ratio may be a marker for an augmented response to genistein [111]. However, in spite of pleiotropic effects of genistein in different breast cancer cell lines, there seems to be an agreement that genistein leads to blockage of the cells in the S and G_2_/M phases [114]. 

Administration of 50 µM apigenin inhibited the proliferation of SK-BR-3 breast cancer cells in correlation with a cell cycle arrest in the G2/M phase induced by increased expression of p21 and p27, and reduced expression of cyclin A, B, D and E and Cdk1 [112]. In conclusion, the major anti-proliferative effect of several polyphenols (quercetin, resveratrol, genistein and apigenin) has been shown to be associated with G2/M phase arrest. 

It was reported in 1998 that 40 µM EGCG inhibited the growth of Hs758T breast cancer cells, but this effect was not observed in their normal counterparts. The reduction in cell density was explained based on the apoptotic effects, demonstrated by terminal deoxynucleotidyl transferase assay [122]. Apoptosis was also induced in MDA-MB-231 breast cancer cells by EGCG (50 and 80 µM) associated with reduced Bcl-2 and Bax expression [115]. Physiological concentrations of EGCG (8 µM) applied in long-term cultures of the same aggressive metastatic breast cancer cell line induced 1.5- to 2-fold higher levels of the basal caspase-3/7 activity [116].

One of the clinical issues after antibody therapy is the development of resistance. Thus, EGCG was investigated for its anti-cancer effects in JIMT-1, a cell line resistant to trastuzumab since its isolation, and in trastuzumab resistant BT474 cells generated by continuous exposure to the antibody. Exposure of the trastuzumab resistant breast cancer cell lines to 80 and 160 µg/mL EGCG for 72 h led to decrease in cell growth and apoptosis at high concentrations associated with decreased Akt activity and induction of Forkhead box O3 transcription factor protein (FOXO3) [123]. The overexpression of 67-kDa laminin receptor (67LR) has been observed in several cancer tissues, including breast cancer, and this phenotype was correlated with tumor progression [124,125]. 67LR was identified as a receptor for EGCG [126] and a modulator of the apoptotic effects induced by the polyphenol [124]. Administration of 75 µM EGCG for 72 h induced late apoptosis/necrosis in SK-BR-3 breast cancer cell line with HER2 overexpression, possibly through 67LR mediated pathway [127]. Not only the EGCG but the other polyphenols can also induce apoptosis. In addition to a block the progression in cell cycle 5 and 10 µM quercetin-3-methyl ether also induces apoptosis evidenced by increased levels of cleaved caspase 3/7 and poly (adenosine diphosphate-ribose) polymerase (PARP) cleavage [100]. Apoptosis was also induced in MCF-7 breast cancer cells by 150 µM quercetin and it was associated with decreased levels of Bcl-2, reduced mitochondrial membrane potential and enhanced level of activated caspase-6, -8, -9 [117]. Induction of apoptosis by 30 and 50 µM resveratrol was dependent on the expression of constitutively active signal transducer and activator of transcription 3 (STAT3) [128]. Experiments with resveratrol in MCF-7 breast cancer cells revealed that the polyphenol acts as an estrogen receptor agonist, but, at the same time, reduces the Bcl-2/Bax ratio implying that it is a candidate for hormone replacement therapy (HRT) [118]. Moreover, treatment with resveratrol induced increased expression of p53, cleavage of PARP and this phenomenon was dependent on nuclear factor erythroid 2 [NF-E2]-related factor 2 (NRF2), which is known as a molecule involved in regulation of the antioxidant response [129].

Recent experiments tested the hypothesis that the inhibition of the IGF1R/Akt/Bcl-2/Bax pathways is responsible for the apoptotic effects of genistein in MCF-7 breast cancer cells. The presence of 40 and 80 µM genistein for 48 h in the cell culture medium induced apoptosis, increased Bax messenger RNA (mRNA) and protein levels, decreased Bcl-2 mRNA and protein levels, decreased insulin growth factor receptor (IGFR) and Akt protein levels [119]. Apigenin was also reported to be involved in apoptosis of breast cancer cells. The inhibition of the proliferation of SK-BR-3 cells by apigenin was correlated with the appearance of a subG0/G1 population, enhanced levels of cleaved caspase-3/8 and PARP, supporting the hypothesis that apigenin induced apoptosis through a caspase-dependent pathway [120]. Moreover, genetically modified MCF-7 cells overexpressing HER2 exhibited apoptosis through the extrinsic pathway, by activation of p53 and inhibition of STAT3 and NF-κB signaling pathways [116]. Recent data support the involvement of curcumin in apoptosis induction as well. Curcumin increased the percentage of breast cancer cells with low level of HER2 in the subG1 population and these data were confirmed by increased Bax/Bcl-2 ratio [121]. Administration of 30 µM curcumin for 48 h induced mainly early apoptosis associated with little late apoptosis in MDA-MB-231 (ER-/PR-/HER2-/EGFR+) breast cancer cells, a model of triple negative breast cancer which is associated with poor prognosis. In MCF-7 breast cancer cells the administration of 12, 24, 36 µM curcumin for 48 h induced early and late apoptosis confirmed by nuclear accumulation of p53 and p21 [109]. To sum up, polyphenols under different environmental conditions may induce processes associated with early or late apoptosis, decreased expression of anti-apoptotic proteins and increased expression of the pro-apoptotic ones. However, most of the concentrations applied in *in vitro* studies are high and this may represent a drawback for clinical applications. Additional studies with physiological concentrations administrated for a longer time or animal experiments to check the toxicity of polyphenols at higher concentrations will help us understand the utility of polyphenols in practice.

### 4.5. Estrogen Receptors and Polyphenols

**Figure 5:**
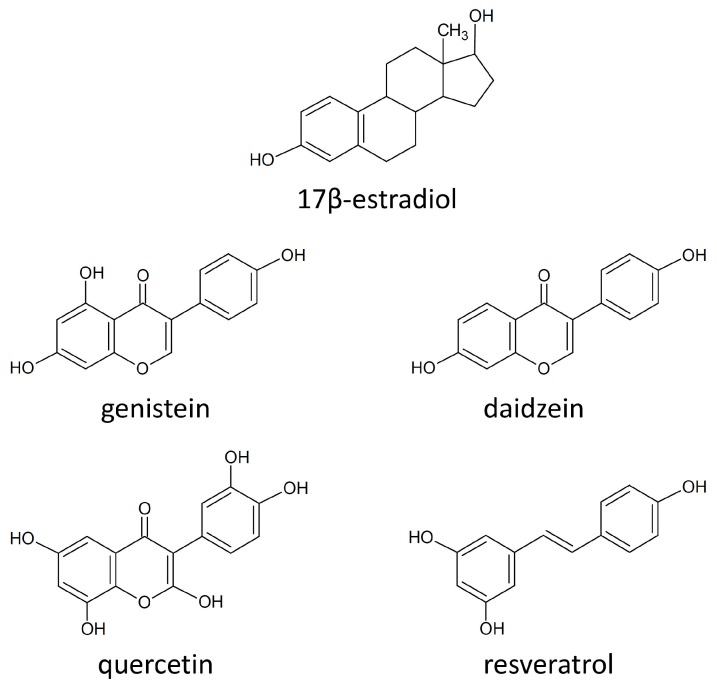
Chemical structure of 17β-estradiol and phytoestrogens [134,138].

Estrogen hormones are thought to be key mediators for the development of the female reproductive system and for the progression of breast cancer [130,131]. Since it was noticed that estrogen administration was associated with breast cancer [132], it has been proposed that phytoestrogens, non-steroidal molecules, due to their structure similar to that of estrogens (Figure 5), could be used for HRT in post-menopausal women and cancer prevention in both pre- and postmenopausal women [2,133]. However, the use of phytoestrogens has not been strongly recommended due to lack of and contradiction between available data [134,135]. Additionally, critical papers considered administration of phytoestrogens contraindicated in patients who survived breast cancer [136]. A possible explanation for the cancer preventive action phytoestrogens/polyphenols is the existence of the two types of estrogen receptors: ERα and ERβ. It was noticed that estradiol particularly binds ERα, while phytoestrogens (like, genistein) bind ERβ. Phytoestrogens rather behave as selective estrogen receptor modulators, with agonistic effects in the uterus and bones, but antagonistic effect in the mammary tissue. Since the function of ERα and ERβ in breast tumor cells was associated with activation and suppression of proliferation, respectively, the selective effect can account for their utility in HRT [137].

**Figure 6:**
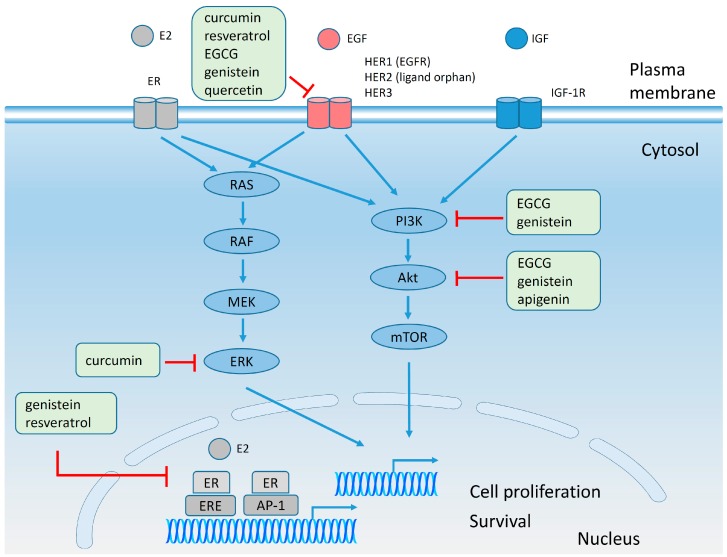
The effect of polyphenols on nuclear ER, HER2 and IGF signaling pathways. Two main mechanisms of E2 action are depicted: (i) genomic activity of nuclear ER in connection with ERE or activating protein-1 (AP-1) transcription factors and (ii) cell surface activity of plasma membrane ER in connection with growth factor receptor signaling pathways [21,139]. Nuclear ER activity might be inhibited by genistein and resveratrol; the expression or phosphorylation of EGFR family can be downregulated by curcumin, resveratrol, quercetin and genistein; ERK phosphorylation can be reduced by curcumin; PI3K activity can be inhibited by EGCG and genistein, while Akt action can be inhibited by EGCG, genistein and apigenin [142,143,144,145,146,147,148,149]. Blue and red lines indicate stimulation and inhibition, respectively (for the sake of simplicity only the major proteins of the signaling pathways were included).

There are several mechanisms of estrogen action at the cellular level (Figure 6): (i) ligand-dependent action through nuclear steroid receptor family; (ii) ligand-independent action through the impact of intracellular kinases on the phosphorylation of nuclear estrogen receptors; (iii) estrogen response element (ERE)-independent action of activated nuclear estrogen receptors on the transcription of genes containing alternative response elements; (iv) non-genomic mechanism trough cell-surface ER connected to intracellular signaling pathways [139]. Cell proliferation and survival can be triggered by ER through genomic activity and in correlation with signaling pathways of receptor tyrosine kinase (RTK) families: EGFR and IGFR. The intracellular signaling pathways responsible for the collaboration between cell-surface ER and RTK are: (i) the mitogen-activated protein kinase (MAPK) pathway including rat sarcoma virus protein homolog (Ras), virus-induced rapidly accelerated fibrosarcoma protein homolog (Raf), MAPK/extracellular signal-regulated protein kinase (ERK) kinase (MEK) and ERK signaling proteins (Ras/Raf/MEK/ERK pathway); (ii) the PI3K/Akt/mammalian target of rapamycin (mTOR) signaling pathway [2,21,35,132,140,141]. 

Some flavonoids like genistein, daidzein and quercetin are known to exert anti-oxidant, anti-tumor and anti-inflammatory effects through their ability to scavenge free radicals [150] and through their capacity to mimic the effect of estrogens in physiological activities [151]. Administration of 10 µM genistein for 6 days bound to estrogen receptors in MCF-7 cells with a dissociation constant (K_d_) of 4 nM and modulated the expression of proteins regulated by ER. Resveratrol influenced the mRNA expression of genes involved in ER signaling pathways with a higher activity in ER+ than ER- breast cancer cells [152]. Additional data about resveratrol indicates that it may activate other pathways independent of ER which are responsible for cell growth inhibition, but it is an ER agonist at low doses (10^−11^–10^−8^ M) [153]. A new theory was put forward by Zeng and coworkers who suggested that up-regulation of ERα in ER negative cell lines may be further utilized to increase sensitivity to anti-hormone therapy [154]. Hence, physiological concentrations of EGCG (0.1–1 µM) were administrated for 48 h to three cancer cell lines and one control breast cell line. In MCF-7 cell line downregulation of ERα was observed, in MDA-MB-231 and T47D cell lines was noticed the increased in ERα, while no detectable changes were identified in MCF-10A control cell line. Further, to confirm their hypothesis, MDA-MB-231 and T47D were treated with EGCG followed by tamoxifen which indeed lead to reduction in cell proliferation [154].

### 4.6. Effect of Polyphenols on Plasma Membrane Receptors and on Signaling Pathways

The effect of polyphenols on intracellular signaling pathways may be a direct effect on kinases and transcription factors or preceded by polyphenol-induced alterations in the activity of membrane receptors (Figure 6). EGCG at concentration of 80 µg/mL was found to inhibit HER2 tyrosine phosphorylation almost completely in mouse mammary tumor virus (MMTV)-HER2 NF639 cell line [144]. Also, exposure of BT474 breast cancer cells to 25 µM genistein for 3 days reduced the expression level of EGFR, HER2 and HER3 [146]. The level of HER2 protein was decreased in a time- and dose-dependent manner in SK-BR-3 breast cancer cells by administration of 100 and 200 µM quercetin due to poly-ubiquitination of HER2 [145]. The inhibitory activity of curcumin was mainly observed on EGFR. It downregulated EGFR and p-EGFR levels in MCF-7 cells [155], reduced the phosphorylation of EGFR in MDA-MB-231 (ER−/PR−/HER2−/EGFR+) breast cancer cells [148] and interrupted the association between α6β4 integrin and EGFR by blocking the distribution α6β4 integrin into lipid rafts [142]. The effect of resveratrol was studied in MCF-7, MDA-MB-231 and SK-BR-3 breast cancer cell lines. In MCF-7 cell line, 10 and 40 µM resveratrol applied for 24 h reduced the cell surface expression of EGFR [143]; in MDA-MB-231 breast cancer cell line resveratrol suppressed EGF-mediated migration and matrix metalloproteinase (MMP)-9 levels [156]; in SK-BR-3 breast cancer cell line resveratrol mediated downregulation of HER2 gene [157]. On the contrary, low concentrations of genistein (1 µM) stimulated the growth of MCF-7 breast cancer cells and increased the expression of IGF-1 receptor. An estrogen receptor antagonist blocked the upregulation of IGF-1 receptor expression induced by genistein, supporting the hypothesis that the effect of genistein on IGF-1 receptor required the communication between IGF-1 receptor and ER pathways [158,159]. The stimulatory effect of genistein may be related to its structural similarity to estrogen and its agonistic effect on estrogen receptor [160]. In addition to the specific effects on membrane receptors polyphenols may alter the structure of lipid rafts. It has been shown that EGCG caused a reduction in the amount of detergent resistant membranes in SK-BR-3 cell line [127].

Cells communicate with each other using signaling molecules which in turn activate intracellular pathways. Most intracellular proteins involved in signaling are kinases which are intensely altered in breast cancer cells and part of these alterations have been related to drug resistance [161,162]. It was shown that EGCG is able to inhibit several signaling pathways. While short-term (24 h) exposure to 40 µg/mL EGCG led to partial inhibition of the PI3K-Akt pathway, promoting cell survival [144], the long-term (two weeks) presence of EGCG in MMTV-HER2 NF639 cell culture medium induced the activation MAPK pathway and resistance to EGCG [163].

The adrenergic system in the human body is associated with stress signaling, which in turn triggers the production of ROS and their production in high quantities can lead to cancer development [164,165,166]. MDA-MB-231 human breast cancer cells expressing β2-adrenergic receptor (β2-AR) were treated with 0.1, 1 and 10 µM quercetin-3-Oglucuronide (Q3G), a circulating metabolite of quercetin, for 24 h. The quercetin metabolite suppressed cAMP production and Ras activation accompanied by a reduction in the level of ROS. All these results suggested that Q3G may be used as a dietary chemo-preventive factor against stress-related breast cancer [167].

Wingless/integration 1 (Wnt) signaling was associated with cancer progression and elevated levels of β-catenin, the central protein responsible for the activation of the canonical Wnt pathway [168,169,170]. The Wnt pathway was suggested to be involved in the epithelial-mesenchymal transition (EMT) and its suppression may inhibit EMT and metastasis [171]. Flow cytometric analysis of MCF-7 breast cancer cells revealed a significant inhibition of Wnt signaling by 10 µM curcumin applied for 12 h [172].

In another breast cancer cell line, MDA-MB-231 (ER−/PR−/HER2−, EGFR+), a model for triple negative breast cancer, administration of 30 µM curcumin for 48 h reduced phosphorylation of ERK [148]. Apigenin was shown to have a multitude of effects on MCF-7 breast cancer cells. It blocked the activities of MAPK, protein kinase A (PKA), p38, Akt suggesting that apigenin might act as a protein kinase inhibitor. Moreover, the administration of 5, 10 and 20 µM apigenin decreased E26 transformation-specific domain-containing protein (Elk1), cAMP response element-binding protein (CREB) and CCAAT-enhancer-binding protein homologous protein (CHOP) levels in MDA-MB-231 breast cancer cells indicating that apigenin may act as an inhibitor of the transcription factors [149]. The potential role of apigenin as an anti-cancer agent was confirmed in HER2-overexpressing MCF-7 cells in which apigenin reduced tyrosine phosphorylation of HER2 and reduced expression of phosphorylated Janus kinase 1 (phospho-JAK1) and phospho-STAT3 [173]. In conclusion, several polyphenols (EGCG, genistein, quercetin, curcumin and resveratrol) successfully reduced the phosphorylation or expression level of EGFR family members in the micromolar concentration range. However, low, physiological concentrations of genistein led to increased IGF1R expression associated with cell proliferation. On the other hand, polyphenols reduced the level of Wnt, a molecule involved in EMT suggesting that they may inhibit the metastastic process. The antiproliferative effect of polyphenols was caused by their ability to inhibit the phosphorylation of key signaling molecules (MAPK, PKA, p38, Akt, Elk, JAK1 and STAT3). Taken together, these results support the pleiotropic effect of natural molecules against malignant transformation.

### 4.7. Epigenetic Mechanisms and Polyphenols

Epigenetic changes are heritable modifications which do not involve changes in the nucleotide sequence of DNA, but still induce alterations in the phenotype. These changes in gene expression may occur during development, differentiation and also may be due to the impact of the environment on the organisms [16,174,175,176,177,178]. At the biochemical level three main epigenetic modifications are known: DNA methylation, histone modification and microRNA (miRNA) expression. *DNA methylation*. Methylation of genes takes place in cytosine-phosphate-guanine (CpG) island of the promoter region leading to silenced gene expression. Five members of DNA methyltransferases (DNMT) exist in mammals: DNMT1, DNMT2, DNMT3α, DNMT3β, and DNMT3L. Tumor progression is regularly correlated with hypermethylation of tumor suppressor genes. *Histone modification.* Positively charged lysine residues in histones are responsible for the attraction between the nucleosomal core and negatively charged DNA leading to condensation of the chromatin (heterochromatin) and inhibited gene transcription. On the contrary, addition of an acetyl group to histones by histone acetylases/histone acethyltransferases (HAT) will remove the positive charge and the chromatin will display a loose structure (euchromatin) which will facilitate gene transcription. Since acetylation is a reversible phenomenon, the reverse process is catalyzed by an enzyme, histone deacetylase (HDAC) which removes acetyl groups from lysine and inhibits gene expression. Abnormal deacetylation of histones due to high activity of HDAC correlated with silencing of the genes was observed in cancer cells. *miRNA expression*. Small regulatory RNA may inhibit protein expression after binding to the target gene. Silencing, down-regulation or dysregulation of miRNAs was reported in breast cancer [16,174,179,180]. Compared to genetic changes the epigenetic modifications are considered reversible [180]. In breast cancer samples DNMT1, DNMT3A, DNMT3B levels were increased 1.8–2.9 fold compared to normal tissue [179], tumor suppressor proteins: cyclin-dependent kinase inhibitor p16 which inhibit Cdk4 (p16INK4a) and alternate reading frame p14 protein (p14ARF) were inactivated [181], while the BRCA1 gene was epigenetically silenced and deleted [182]. The expression of several DNA methyl transferases (DNMT1, DNMT3a, and DNMT3b) has been found to be elevated in breast cancer tissue. Since several natural compounds (EGCG, genistein, withaferin A, curcumin and resveratrol) have been reported to decrease this elevated transcription of DNMT1, DNMT3a, and DNMT3b genes, the lower incidence of breast cancer among Asian women, who consume more of these natural compounds, may be related to the demethylation potential of these polyphenols [16,183].

Normal cells undergo senescence by losing 150–300 bp from their telomeres with each cell division. In cancer cells this phenomenon is avoided by enzymes called telomerases. Telomerases have a low activity in normal cells, but they were identified as being highly active in 90% of cancers. The catalytic subunit of telomerase is human telomerase reverse transcriptase (hTERT). The actual paradigm which supports the idea that promoter hypermethylation represses gene transcription [184] was challenged by the observation that hypermethylation of hTERT promoter was associated with increased expression of telomerase in cells [185,186]. EGCG treatment of MCF-7 cells inhibited the activity of DNMT1 leading to hypomethylation of hTERT followed by reduced hTERT transcription [187]. Similarly, genistein inhibited the transcription of hTERT, downregulated DNMT1, DNMT3a, DNMT3b [188]. Although much less data is available about the effect of polyphenols on miRNA (miR) expression, it has been reported that 10 to 60 µM curcumin upregulated miR-15 and miR-16 transcript levels in MCF-7 cells associated with reduced expression of Bcl-2, one of the anti-apoptotic proteins [189]. In conclusion, polyphenols like EGCG, genistein, curcumin and resveratrol downregulated the expression of DNA methyl transferases in breast cancer cell lines. Nevertheless, the scarcity of the data urges us to investigate the epigenetic modifications induced by polyphenols further, particularly since epigenetic changes are reversible compared to genetic modifications considered to be irreversible processes.

### 4.8. Breast Cancer Stem Cells (BCSC) and Polyphenols

The concept of cancer stem cells suggests that certain tumor cells are capable of self-renewal similar to normal proliferative tissues (skin epithelium, intestinal epithelium or bone marrow) [190]. Opposed to leukemic stem cells which can be identified by markers such as CD34 and CD38, cancer stem cells from solid tumors are poorly characterized. CD24^−^ and CD44^+^ cells have been putatively identified as breast cancer stem cells [190,191]. The anti-cancer effects of resveratrol were studied in CD24^−^/CD44^+^/epithelial specific antigen (ESA)^+^ populations of cancer stem cells selected from MCF-7 and MDA-MB-231 breast cancer cell lines. Exposure of the breast cancer stem cells to 50 and 100 µM resveratrol for 72 h reduced cell viability and mammosphere formation, induced apoptosis and reduced lipid synthesis confirmed by down-regulation of fatty acid synthase (FAS) [192].

Administration of 40 to 160 µg/mL EGCG induced cell death and reduced mammosphere formation in stem-like SUM-149 cells selected from SUM-149 inflammatory breast cancer cell lines associated with very aggressive phenotype [193]. Cancer stem cells with a CD44^+^/CD24^−/low^ phenotype were isolated from two other breast cancer cell lines (MCF-7 and T47D). Exposure of breast cancer stem cells to 5 to 20 µM curcumin for 24 h inhibited migration and mammosphere formation, increased the expression of the epithelial markers cytokeratin 18 and 19, while decreasing the expression of Cyclin D1, avian myelocytomatosis viral oncoprotein homolog (c-myc), vimentin, MMP-2,-9 and the nuclear localization of β-catenin [194]. These results suggest that polyphenols have an inhibitory effect on cancer stem cells.

### 4.9. EMT and Polyphenols

Epithelial mesenchymal transition has been described as a process in which cells in normal and tumor tissues migrate and invade other tissues. At the beginning of EMT the expression of the epithelial markers (E-cadherin and γ-catenin) is downregulated, while mesenchymal markers such as MMP-2, and -9, fibronectin, vimentin are up-regulated. The entire transformation of the cells will provide them with a new phenotype which will allow them to migrate and invade the surrounding environment, an essential step in metastasis formation. After reaching the target tissue, the cells will undergo a reversal process, a mesenchymal-epithelial transition (MET) characterized by the upregulation of epithelial markers [195,196]. Exposure to 20 µM curcumin for 48 h decreased the expression of vimentin, increased the expression of E-cadherin, inhibited cell motility and invasiveness in MCF-7, MDA-MB-231 breast cancer cells exposed to lipopolysaccharide (LPS) in order to trigger EMT [197]. Exposure of MCF-7 breast cancer cells to environmental carcinogens: 4-(methylnitrosamino)-1-(3-pyridyl)-1-butanone (NNK), benzo[a]pyrene (B[a]P) and 2-amino-1-methyl-6-phenylimidazo[4,5-b]pyridine (PhIP) increased the expression of the EMT markers MMP-9 and vimentin while diminishing the level of E-cadherin. Treatment of the transformed cells with 10 and 20 µM EGCG attenuated EMT characteristics [47]. Dimethylbenz[a]anthracene (DMBA)-induced mouse mammary adenocarcinoma cell lines displayed decreased level of E-cadherin which was up-regulated by 60 µg/mL EGCG [198]. The EMT phenotype in EGF-treated MCF-7 cells characterized by low E-cadherin, γ-catenin and increased vimentin, fibronectin, N-cadherin expression was normalized and enhanced migration was inhibited by treatment with resveratrol [199,200]. It can be concluded that polyphenols revert the process of EMT and are, therefore, expected to decrease the metastatic potential of cancers.

### 4.10. Administration of Polyphenols as Nanoparticles

Since the toxicity of chemotherapy requires strategies to reduce its side effects, administration of conventional chemotherapeutic agents in nanoparticles and their combination with polyphenols emerge as a novel modality in breast cancer therapy. Moreover, the low level of stability of polyphenols compromising their bioavailability requires a new approach to increase intracellular stability and constant release of the compounds [201]. In a recent, complex study Narayanan and coworkers observed that combination nanoparticles containing EGCG and paclitaxel increased apoptosis, inhibited NF-κB activation, down-regulated the major genes involved in metastasis, angiogenesis and cell survival in MDA-MB-231 cells. These effects were significantly enhanced when the nanoparticles were targeted with anti-EGFR antibodies. At the same time, multidrug resistance developed in MDA-MB-231 cells was inhibited by EGCG-containing nanoparticles shown by downregulated P-glycoprotein expression. Moreover, the effect of combination nanoparticles containing EGCG and paclitaxel was tested in samples from patients with breast cancer and their effectiveness was found to be correlated with high Ki-67 proliferation index [201]. The stability, sustained release, intracellular concentration of EGCG was increased if it was loaded into liposomes or chitosan-coated liposomes. Both types of nanoparticles were superior to free EGCG in reducing cell viability and inducing apoptosis [202]. Encapsulating quercetin in methoxypolyethylene glycol-polylactic acid (MPEG-PLA) is a modality to defeat its hydrophobicity. Quercetin nanoparticles with 155 nm size induced apoptosis in a triple negative cell model of breast cancer, MDA-MB-231 cell line, with the drug being released for 10 days [203]. An improved system of targeted nanoparticles with polyphenols displaying higher selectivity was presented by Catania and coworkers who showed that curcumin and resveratrol-containing liposomes coupled to anti-HER2 antibodies had enhanced cellular uptake, cytotoxic and antiproliferative effects mainly in the breast cancer cell line with the highest expression of HER2 [204]. In order to increase the stability and longer retention of polyphenols inside of the cells, curcumin and polylactic-co-glycolic acid (PLGA) nanoparticles were prepared. The exposure of MCF-7 cells to nanoparticles inhibited cell proliferation, induced apoptosis, released curcumin for 10 days *in vitro* and blocked the cell cycle in the S and G2/M phases [205]. The results beginning to emerge establish that encapsulation of polyphenols into nanoparticles increases their bioavailability and effectiveness. However, additional data are required in order to demonstrate that polyphenols reduce the cytotoxicity of conventional chemotherapy in breast cancer cells.

### 4.11. Combined Applications of Polyphenols in Vitro

Polyphenols have multiple beneficial effects in cardiovascular and neurodegenerative diseases as well as in cancer, but their poor bioavailability together with rapid degradation, metabolization and excretion is a significant obstacle to their successful application. This drawback can be overcome by combining different polyphenols or to associate polyphenols with allopathic therapy [28]. The effect of combined EGCG and curcumin was studied in doxorubicin resistant MCF-7 breast cancer cells. EGCG alone induced growth inhibition, apoptosis, while curcumin alone inhibited the function of P-glycoprotein. Exposure to the combination of EGCG and curcumin enhanced the toxicity of doxorubicin in MCF-7 cells [206]. Synergistic cytotoxic effects and arrest of the cells in the G2/M phase was observed for the same polyphenol combination in MDA-MB-231 breast cancer cells [207]. TNBC is notorious for its therapy resistance [208,209,210,211]. A dual approach, targeting mutant p53 by siRNA and administration of EGCG, a polyphenol frequently reported to have pro-apoptotic properties, has been studied in a TNBC cell line (Hs578T) expressing mutant p53. The combination of p53 small interfering RNA (siRNA) and EGCG increased apoptosis more than any treatment alone. The results reinforced the idea that multi-targeted therapy will enhance the anti-cancer effect in a cell line model of TNBC [212]. Administration of resveratrol, Herceptin and combination of resveratrol with Herceptin was investigated in MCF-7 and T47D breast cancer cell lines. Combination of resveratrol and Herceptin reduced cell growth and HER2 expression in both cell lines and increased subG1 fraction compared to control samples or each treatment applied alone [213]. However, administration of resveratrol decreased cell death induced by paclitaxel in MDA-MB-231 and SK-BR-3, but not in MCF-7 breast cancer cells [214]. Attention must be paid to genistein in combination with other drugs. The combinatorial effect of cisplatin, paclitaxel or tamoxifen with genistein was studied in breast cancer cell lines with different ERα/ERβ ratio: MCF-7 (high ratio), T47D (low ratio), MCF-7 overexpressing ERβ. Combination of cisplatin with genistein or tamoxifen with genistein decreased ROS production, apoptosis and autophagy in MCF-7 cells, but not in T47D and MCF-7 overexpressing ERβ, suggesting that in breast cancers with high ERα/ERβ ratio administration or consumption of genistein may be harmful [111].

## 5. *In Vivo* Experiments

### 5.1. The Effect of Polyphenols on Tumor Growth—Animal Models

Since the administration of hormones in HRT may promote late stages of carcinogenesis in postmenopausal women, different strategies have been proposed to alleviate postmenopausal symptoms, to inhibit osteoporosis and to prevent heart diseases and a possible solution might be the administration of natural compounds [130]. However, the inconsistency and contradictory results should warn us to interpret these data carefully [136,215].

Earlier *in vivo* result communicated the beneficial effect of genistein administrated to pre-pubertal Sprague-Dawley rats with DMBA induced tumors; accordingly, reduction in carcinoma incidence, reduction in tumor multiplicity, upregulation of BRCA1 mRNA was reported in correlation of genistein administration in rats with chemical induction of breast carcinoma [216,217]. In a transgenic mouse model of breast cancer (mouse mammary tumor virus-neu (MMTV-neu) transgenic mice) administration of genistein decreased the mammary tumor latency compared to control group [217]. On the other hand, an entire series of papers published by Helferich and co-workers warn us about the effects of genistein in ovariectomized athymic mice. The data from these papers reported increased tumor size in dose-dependent manner, increased cell proliferation and increased expression of pS2, an estrogen responsive gene; moreover, genistein annulated the effect of tamoxifen and increase progesterone and cyclin D1 levels [218,219]. Likewise, adult female Sprague-Dawley female rats with chemical induction of breast tumors, after the exposure to genistein displayed increased tumor cross-sectional area, increased tumor multiplicity, but no effect on tumor incidence compared to control rats [220]. The data reported about administration of genistein in animal models of breast cancer are contradictory (Table 3), since earlier papers indicated a beneficial effect of genistein, while later publications associate administration of genistein with increased incidence of breast cancer. These inconsistencies might be explained by various factors: (i) the animal models reported are highly different, from chemically-induced breast cancer in rats to transgenic mice or nude mice with human xenograft tumor; (ii) the doses and the periods used for the administration of genistein are highly heterogeneous and make the reports difficult to be compared; (iii) each experiment report other output parameters; and (iv) the toxicity of the polyphenols was not always taken in account.

**Table 3:** Summary of *in vivo* experiments: breast cancer and polyphenols.

Author, Year	Animals	Dose and Duration of Administration	Result
**Genistein**			
Murrill W.B. *et al.*, 1996 [216]	Pre-pubertal Sprague-Dawley rats with DMBA induced carcinoma	500 μg/g body weight in P16, P18, P20	Reduction in carcinoma incidence
Jin Z. *et al.*, 2002 [217]	Pre-pubertal rats with DMBA induced carcinoma	500 mg/kg body weight in P7, P20	Reduction in tumor multiplicity by 60%
Cabanes A. *et al.*, 2004 [221]	Pre-pubertal female rats with DMBA induced carcinoma	50 µg (injection) daily from P7 to P20	Reduction in the size of the mammary epithelial area, reduction in number of TEB, increased density of lobulo-alveolar structures (increased differentiation), up-regulation of breast cancer tumor suppressor gene 1 (BRCA1) mRNA
Ju Y.H. *et al.*, 2001 [218]	Ovariectomized athymic mice with MCF-7 xenografts	125, 1000 μg/g body weight in the diet for 22 weeks	Tumor size was increased in dose-dependent manner; cell proliferation was enhanced at concentration >250 μg/g; increased in pS2, an estrogen responsive gene at concentration >500 μg/g
Ju Y.H. *et al.*, 2002 [219]	Ovariectomized athymic BALB/c (nude) mice with MCF-7 xenografts	1000 ppm (1000 μg/g body weight)	Genistein canceled the inhibitory effect of tamoxifen, decreased estradiol level in plasma, increased expression estradiol regulated genes (pS2, progesterone, cyclin D1)
Jin Z. *et al.*, 2002 [217]	Transgenic mice for MMTV-neu gene	250 mg/kg for 7 weeks	Mammary tumor latency delayed compared to controls; no reduction in in the number or tumor size
Kijkuokool P. *et al.,* 2006 [220]	Adult female Sprague-Dawley rats exposed to NMU	1 mg/kg body weight daily subcutaneous injection for 20 weeks	Increased tumor cross-sectional area, increased tumor multiplicity, but not tumor incidence
**Daidzein**			
Constantinou A.I. *et al.*, 2001 [222]	Female Sprague-Dawley rats with DMBA breast carcinoma induction	200 mg/kg diet	Tumor incidence and survival similar to control groups; reduction in tumor multiplicity by 32%; increased median tumor latency
Jin Z. *et al.*, 2002 [217]	Transgenic mice for MMTV-neu gene	250 mg/kg for 7 weeks	Mammary tumor latency delayed compared to controls; no reduction in in the number or tumor size
Lamartiniere C.A. *et al.*, 2002 [223]	Virgin female rats	250 and 1000 mg/kg in the diet, 2 weeks prior to breeding till 50 day postpartum	Moderate reduction in ovarian and uterine weights and mammary gland size; reduced body weight; reduction in circulating progesterone
Ju Y.H. *et al.*, 2006 [224]	Ovariectomized athymic mice with MCF-7 human xenografts	125 to 1000 ppm (125 to 1000 μg/g body weight)	No statistical significant reduction in tumor size and proliferation
**Resveratrol**			
Banerjee S. *et al.*, 2002 [225]	Female Sprague-Dawley rats with DMBA breast carcinoma induction	10 ppm	Reduction in the incidence (by 45%) and multiplicity (by 55%) of the tumors; increased latency period; suppressed COX-2, MMP-9, NF-kB; no effect on body weight or tumor volume
Whitsett T. *et al.*, 2006 [226]	Female Sprague-Dawley rats with DMBA breast carcinoma induction	1 g/kg in the diet	Suppression of mammary carcionogenesis: less number of tumors per rat, longer tumor latency; reduced proliferation; increased apoptosis in epithelial cells of TEB; reduced toxicity: no alterations in body weight
Singh B. *et al.*, 2014 [129]	August Copenhagen Irish rats (rodent model of breast carcionogenesis)	50 mg subcutaneous pellet per month, 8 months	Decreased tumor incidence and increases latency in mammary tumors induced by estradiol; upregulated NRF2, a regulator of the anti-oxidant response; induced apoptosis (increased p53 and PARP cleavage) in mammary tissue
**EGCG**			
Whitsett T. *et al.*, 2006 [226]	Female Sprague Dawley rats with DMBA and NMU mammary cancer induction	0.065% in the drinking water	Not efficient in reduction of breast cancer incidence at these doses
**Quercetin**			
Verma A.K. *et al.*, 1998 [227]	Female Sprague Dawley rats with DMBA and NMU mammary cancer induction	5% in the diet	Reduction in the number of tumors; decreased tumor multiplicity; no detectable signs of toxicity (similar body weight in treated and control rats)
Singh B. *et al.*, 2010 [228]	Female August Copenhagen Irish (ACI) rats	2.5 g/kg in diet, 8 months	No induction of tumors in ACI rats; did not protect against estrogen-induced tumors; did not confer protection against breast cancer and may worsen breast cancer status regularly exposed to estradiol
**Curcumin**			
Masuelli L. *et al.*, 2013 [121]	BALB-neuT transgenic mice for neu oncogene	n.m.	Increased tumor-free survival; reduction in tumor multiplicity; safe to be administrated: no modification in hematological and clinical chemistry parameters

Abbreviations: P, postnatal day; DMBA, 7,12-dimethylbenz(a)antracen; TEB, terminal end buds; BRCA1, breast cancer tumor suppressor gene 1; Nrf2, nuclear factor-erythroid 2-related factor-2; NMU, *N*-methyl-*N*-nitrosourea; ppm, parts per million; MMTV, mouse mammary tumor virus; n.m., not mentioned.

Both identification/generation of the animal models comparable to human disease and systematic investigation of parameters still remain a challenge for the scientific world. The effect of daidzein, another polyphenol with phytoestrogen characteristics, was investigated in different animal models ranging from breast cancer in mice transgenic for neu oncogene to chemically induced breast cancer and human xenograft tumors. In Neu-transgenic mice, mammary tumor latency was delayed by daidzein and a 32% reduction in tumor multiplicity was noticed in chemically induced breast cancer [222]. However, administration of daidzein in ovariectomized athymic mice induced no significant changes in tumor size and proliferation [224]. The toxicity of daidzein on the reproductive tract of virgin female rats was investigated by Lamartiniere and co-workers. The authors reported moderate reduction in ovarian and uterine weights, slight reduction in mammary gland size and decreased levels of circulating progesterone [223].

The experiments on female Sprague-Dawley rats with chemical breast cancer induction demonstrated that administration of resveratrol reduced the incidence and multiplicity of the tumors, increased the latency period, suppressed COX-2, MMP-9 and NF-κB levels, increased apoptosis in epithelial cells and did not modify the body weight [225,226]. Administration of resveratrol as a subcutaneous pellet for eight months in a rodent model of breast carcionogenesis (August Copenhagen Irish rats) decreased the tumor incidence, increased tumor latency and increased apoptosis in mammary tissue [129]. Low doses of EGCG displayed reduced efficiency against breast cancer in female Sprague-Dawley rats with chemical breast cancer induction [226].Former data about administration of quercetin to female Sprague-Dawley rats with chemical induction of breast cancer reported reduction in the number of tumor, decreased tumor multiplicity and no detectable signs of toxicity [227]. Nevertheless, more recent data reported that quercetin did not confer protection against breast cancer and even worse, it might increase tumorigenesis in animals with chronical exposure to estrogen [228]. Recent data reported that curcumin increased tumor-free survival, reduced tumor multiplicity and displayed no toxicity in BALB-neuT transgenic mice [121]. 

In conclusion, the majority of evidence supports the cancer-preventive effects of polyphenols, although contradictory results have also been published.

### 5.2. Involvement of Polyphenols in Modulation of Metastasis and Angiogenesis

The overall survival rate of patients with breast cancer has improved as a result of early detection of the disease [23]. However, dormant tumor cells are responsible for the development of distant metastases or recurrent disease in 25%–45% of patients 10–15 years after the patient was considered to be cured [22,23]. Data from animal models for metastasis treated with polyphenols offered some promising results. The administration of resveratrol, quercetin, catechin (5 mg/kg) to nude mice with MDA-MB-435 mammary tumors reduced tumor growth and metastasis formation [229]. In another model of mouse breast cancer, BALB/c mice bearing 4T1 breast tumors were exposed to resveratrol for 21 days. The polyphenol decreased the number of pulmonary nodules and the plasma level of MMP-9 [230]. Genistein reduced the number of lung metastases 10-fold in a preclinical xenograft model of breast cancer metastasis in which MDA-MB-435 human breast carcinoma cells were implanted in the mammary fat pad of female nude mice (orthotopic tumor model) [231]. Data reported very recently shed light on the effect of dendrosomal curcumin (supramolecular structures which encapsulates repetitive branched molecules) in mice with 4T1 tumors. Exposure to 80 mg/kg dendrosomal curcumin significantly increased the survival rate and decreased metastasis formation accompanied by downregulated expression of NF-κB, VEGF, COX-2 and MMP-9 in the breast tumor as well as in lung, brain, spleen and liver tissues [232]. Similarly, mice subcutaneously inoculated with 4T1 mouse breast cancer cells were exposed to curcumin loaded into biodegradable micelles. Curcumin inhibited the tumor growth and the formation of spontaneous pulmonary metastases, prolonged survival of the mice and reduced angiogenesis [233]. However, Helferich’s group warned us about the involvement of soy isoflavones in promoting the development of breast cancer [234]. In an attempt to model bone micrometastasis female BALB/c mice were injected intra-tibially with 4T1 mouse mammary cancer cells. A combination of 750 mg/kg soy isoflavones (genistein, daidzein and equol) administrated 3 weeks before and after tumor inoculation increased the bone microtumors and stimulated the formation of lung metastases [234]. In summary, although several lines of evidence suggest that polyphenols can decrease the number of metastases, there are some data contradicting these observations. Therefore the administration of polyphenols for metastasis prevention should be carefully designed and the expected results double checked.

The size of tumors cannot extend beyond 1–2 mm without a proper vascular network [235]. Several modalities which trigger and support the angiogenetic process have been described, starting from sprouting angiogenesis and vasculogenesis to the development of tumor cells which can mimic endothelial cells or the generation of the endothelial cells originating from cancer stem cells [236]. VEGF family of growth factors and hypoxia have been identified as the main factors responsible for the angiogenetic processes [237]. In order to block angiogenesis several drugs have been approved: bevacizumab (Avastin), an antibody directed against VEGF and tyrosine kinase inhibitors (TKI) of VEGFR, such as sorafenib and sunitinib [236,238]. Nevertheless, the side effects of antibody and TKI treatment are difficult to control and some of the patients are refractory to these therapies [236]. In light of the complex and controversial effects of available drugs research into the possible anti-angiogenic effects of EGCG are warranted. Administration of EGCG (50–100 mg/kg/day) for 4 weeks reduced tumor weight, decreased capillary density in the tumors, diminished tumor VEGF expression without affecting body weight and angiogenesis in the heart in C57BL/6J mice with E0771 mouse breast cancer cells implanted in the mammary gland fat pad [239]. However, the same polyphenol did not influence microvessel density in a different animal model [240]. The influence of soy isoflavones on angiogenesis was investigated in animal models and exposure of DMBA-induced mammary tumors to genistein reduced microvascular density and plasma VEGF and increased plasma levels of endostatin, an anti-angiogenic agent [241]. Likewise, 10 mg/kg/day genistein reduced angiogenesis in mice with F3II mammary cancer cells [242]. The angiogenesis in nude mice with MDA-MB-231 breast cancer cells was inhibited by resveratrol [243] and by curcumin [244]. To sum up, although there are some slight contradictions, polyphenol treatment can reduce angiogenesis most of the time thereby slowing down tumor growth.

### 5.3. Combined Administration of Polyphenols–In Vivo Studies

The beneficial effect of combining polyphenols with conventional drugs in *in vitro* experiments laid the foundation for testing such treatments in animal models. Administration of Herceptin and genistein in combination to athymic mice with BT474 breast tumor cells injected subcutaneously decreased the tumor size to the same level as Herceptin alone, suggesting that genistein did not improve the outcome in this type of experiment [245]. Nude mice with MCF-7 cells implanted in the mammary fat pad were treated with genistein, tamoxifen and a combination of genistein and tamoxifen. Reduction in tumor growth, increase in apoptosis index, decrease in proliferation index, reduction in the number of vessels in tumors and decrease in estradiol levels were associated with synergistic activity of genistein and tamoxifen. Additionally, administration of soy phytochemical concentrate in combination with tamoxifen amplified the above mentioned effects, suggesting that soy compounds might be used in prevention or/and treatment of estrogen breast cancers [146]. In contrast with the previous data, Helferich and co-workers reported that inhibitory effect of tamoxifen in ovariectomized athymic mice with MCF-7 xenograft tumors was abolished by genistein in the presence of low levels of estrogen. This finding was confirmed by investigation of the combinatorial effect of estrogen, tamoxifen and genistein on xenograft tumors in nude mice. Combinatorial administration of estrogen, tamoxifen and genistein increased proliferation, decreased apoptosis, increased the levels of cyclin D1 and progesterone receptors mRNA, suggesting that genistein consumption should be recommended with precaution in breast cancer patients receiving tamoxifen [246]. However, a recent review about the effect of soy on breast cancer in preclinical studies suggested that it is premature to rush any conclusions about stimulatory effect of isoflavones on breast cancer and about their ability to abolish the effect of tamoxifen without understanding their mechanism of action at the molecular level. Identification of the molecular targets of soy isoflavones in human disease will be necessary before reaching any solid conclusions regarding the chemopreventive or therapeutic effect of soy consumption in breast cancer [247].

Immunodeficient mice bearing patient-derived trastuzumab- and lapatinib-resistant HER2+ breast tumors revealed that such refractory tumors respond well to the combination of pertuzumab and EGCG suggested by a considerable reduction in tumor size and apoptosis identified in tumor sections by the terminal deoxynucleotidyl transferase dUTP nick end labeling (TUNEL) method. The authors attribute the synergistic effect of adding EGCG to the treatment protocol to its ability to block fatty acid synthase [248]. Breast cancer xenografts injected into the mammary fat pad of athymic female mice exposed to combined resveratrol, quercetin, catechin treatment responded well to the combined therapy [249]. Combined administration of EGCG and curcumin (25 mg/kg/day and 200 mg/kg/day, respectively) in athymic female mice implanted with MDA-MB-231 cells decreased tumor volume and reduced VEGFR-1 expression in tumors [207]. Administration of EGCG in combination with taxol in BALB/c mice injected subcutaneously with 4T1 mouse breast cancer cells significantly decreased tumor growth and the number of lung metastasis, while exposure to EGCG or taxol alone had no significant effect [250]. Another line of evidence for the efficiency of the combination of paclitaxel and polyphenols was presented by Kang and coworkers who showed that paclitaxel and curcumin decreased tumor cell proliferation, increased apoptosis and decreased expression of MMP-9 in a breast cancer murine model with MDA-MB-231 cells [251]. However, administration of resveratrol after paclitaxel treatment suppressed tumor cell death in athymic mice with MDA-MB-231 xenografts [214]. The results of experiments applying polyphenols in combination *in vivo* are in agreement with *in vitro* studies and imply that polyphenols usually increase the efficiency of conventional drugs without enhancing toxicity.

## 6. Clinical Implications of Polyphenols

### 6.1. Bioavailability of Polyphenols in Human Body

Bioavailability, according to the Federal Food, Drug and Cosmetic Act in the US represent “the rate and extent to which the active ingredient or active moiety is absorbed from a drug product and becomes available at the site of action” [252]. The mode of application must also be considered when characterizing bioavailability. In the case of intravenous administration only the ability of the host organism to metabolize the nutrient must be taken into account, while both metabolic and digestive processing must be considered in the case of oral application [253,254]. The bioavailability of polyphenols in humans is explained in great detail in two reviews published by Claudine Manach and co-workers [30,255]. Table 4 presents the sources, the used doses, the plasma concentration and the elimination half-life of polyphenols in the human body.

**Table 4:** Bioavailability of main polyphenols in the human body (adapted from Manach C. *et al.*, [255]).

Source/Polyphenol	Dose	Concentration in Plasma (µM)	Half-Life (h)	Ref.
Onions	100 mg quercetin eq	7.6	10.9	[256]
Apples	107 mg quercetin eq	0.3	23.0	[257]
Quercetin	50 mg	0.29	15.0	[258]
Orange juice	126 mg hesperitin eq	2.2	2.2	[259]
Orange juice	23 mg narigerin eq	0.64	1.3	[259]
Grapefruit juice	199 mg narigerin eq	5.99	2.2	[259]
Chocolate (80 g)	164 mg epicatechin eq	0.7	1.9–2.3	[260]
Red wine (120 mL)	35 mg catechin eq	0.091	3.1	[261]
EGCG	800 mg	2.33	1.9–4.6	[262]
Soy beverage	0.6 mg/kg daidzein eq	0.3	3.4	[263]
Soy beverage	1 mg/kg genistein eq	0.65	7.9	[263]
Daidzein	50 mg	0.76	9.3	[264]
Genistein	50 mg	1.26	6.8	[264]

Additional aspects should be taken into account when the availability of polyphenols in the human body is discussed, since (i) in both *in vitro* and *in vivo* experiments the concentrations of polyphenols are much higher than those reached in the biological target when dietary uptake of polyphenols in humans is considered; (ii) the anti-cancer effects may be due to the glycosylated or methylated metabolic products of polyphenols and not only to the aglycone alone; (iii) urinary excretion of polyphenols may reflect their bioavailability, but also excretion through the bile must be taken into account, e.g., in the case of EGCG and genistein; (iv) generation of some of the polyphenol metabolites with anti-cancer effects depends on the microbial intestinal metabolism, dividing individuals into “producer” and “non-producer” phenotypes; (v) some of the polyphenol metabolites display more pronounced anti-cancer effects than the aglycone itself (*i.e.*, equol, the metabolite of daidzein) [30,255].

### 6.2. Chemoprevention of Breast Cancer by Polyphenols in Humans

At the end of the review we would like to consider the cancer-preventive effects of dietary polyphenols (Table 5). Since results of studies with genistein, the polyphenol studied the most, are contradictory, three, strikingly different hypotheses have been put forward for the effect of soy diet in humans: (i) there is an inverse correlation between soy intake and breast cancer; (ii) soy isoflavones do not change the evolution of breast cancer; (iii) on the contrary, genistein may have the ability to increase the proliferation of breast cancer tumors. The first proposition assuming a favorable relationship between increased soy consumption and decreased breast cancer risk is supported by the strongest lines of scientific evidence. The intake of soy food in adolescence (13–15 years old) by Asian girls may reduce the risk of breast cancer during adulthood, since the breast tissue in adolescence is exposed to various changes and becomes more sensitive [265]. An epidemiological study of 501 breast cancer patients and 594 controls indicated that the intake of soy food in adolescence (at least once per week) and in adult life was inversely and dose-dependently correlated with the risk of breast cancer [265]. High soy consumption was associated with a slight reduction in breast cancer risk with better results in premenopausal women compared to postmenopausal ones (evidence from 18 epidemiological studies) [215]. The conclusion regarding the relationship between soy consumption and breast cancer seemed to be different between women in Asian and Western countries. Thus, soy intake had minor protective activity in post-menopausal women from Western countries, while in pre- and postmenopausal women in Asian countries it showed a protective effect [266,267]. However, some reports support the association between high plasma levels of circulating genistein and reduction of breast cancer in the Dutch population [268]. The decreased risk of breast cancer was associated with two types of diet: (i) Japanese/Chinese diet (2–3 meals/day with 25–50 mg isoflavone each) with the recommendation of more than 100 mg isoflavones in the case of breast cancer patients, since there is no evidence for the adverse effect of soy consumption [269,270]; (ii) Mediterranean diet with the recommendation of vegetables, fruits, fish and soy intake [271]. Attention must be paid to high consumption of fat and alcohol, since both aliments increased the risk of breast cancer [270,271].

The following breast cancer markers have been considered valuable for the evaluation of the effect of dietary polyphenols in humans: plasma hormone levels, breast tissue density (mammography), proliferation index and estrogen concentration at the tumor site [272,273]. Nevertheless, the use of biomarkers from breast tissue collected by fine-needle aspiration (proliferation index/Ki-67 and concentration of hormones in the tumor) are more reliable than so called “surrogate” markers like plasma hormone level and breast tissue density [274]. Still, additional biomarkers for the modifications induced by isoflavones in breast cancer (genetic, metabolic, magnetic resonance imaging) are necessary [138].

**Table 5:** Summary of the association between breast cancer risk and soy intake.

Author, Year	Date of Study	Cases ^1^	Ctrl ^1^	Diet	Dose ^2^	OR/HR/RR (95% CI)	Conclusion
Liu X.O. *et al.*, 2014 [270]	1990–2013	9299	11,412	Soy (soy protein, soy food, soya- bean milk)	1–8 times/week 12.9–500 mg/day ^3^	0.65 (0.43–0.99)	Soy intake was associate with reduction in breast cancer risk (Chinese women)
Nagata C. *et al.*, 2014 ^a^ [267]	1985–2005	2531	25,332	Soy (tofu, soybeans, miso soup)	1–3 times/week	0.62 (0.38–1.01) to 1.59 (0.90–2.81)	Soy intake was associated with moderate and strong reduction of breast cancer in post-menopausal Japanese women
Fritz H. *et al.*, 2013 ^b^ [269]	1992–2012	1830 ^c^	n.m.	Soy (soy food, soy protein, genistein, IF)	Soy 13.03–65.7 g/day IF 7.48–62.68 mg/day	0.25 (0.10–0.61) to 1.19 (0.76–1.85)	Soy intake was associated with no change and increase survival, no change and decrease recurrence of breast cancer in Chinese, Korean, USA, Shanghai women, respectively
Guha N. *et al.*, 2009 [275]	1997–2000	1954 ^d^	n.m.	Daidzein, genistein, glycetin	Daidzein 1.5–9.6 mg/day 0.1–7.8 µg/day Genistein 2.2–13 mg/day 0.1–7 µg/day Glycetin 8.2–15 µg/day 3.6–8.2 µg/day	Daidzein 0.71 (0.45–1.11) to 1.16 (0.81–1.68) Genistein 0.72 (0.46–1.13) to 1.09 (0.76–1.58) Glycetin 0.68 (0.46–1.01) to 1.01 (0.71–1.43)	Decreased risk of breast cancer recurrence was associated with high daidzein and glycetin intake in postM women Women treated with Tamoxifen presented 60% decrease in breast cancer recurrence after daidzein intake
Trock B.J. *et al.*, 2006 ^a^ [215]	1978–2004	7453	16,521	Soy protein and tofu	1–5 times/week 1.6–3.5 g/day	0.86 (0.75–0.99) preM 0.70 (0.58–0.85) postM 0.77 (0.60–0.98)	Increased soy intake was associated with modest reduction in breast cancer risk Inverse association between soy exposure and breast cancer risk in preM (“stronger”) and postM women Caution with interpreting the data due to high heterogeneity of soy exposure
Wu A.H. *et al.*, 2002 [276]	1995–1998	501	594	Tofu—adolescence IF—adult	1–3 times/month +4 times/week >1.79–6.24 mg/1000 kcal >12.68 mg/1000 kcal	0.75 (0.48–1.15) 0.51 (0.31–0.84) 0.76 (0.53–1.09) 0.51 (0.33–0.78)	High soy intake during adolescence and adult life was associated with reduced risk of breast cancer (Chinese, Japanese, Filipino women in Los Angeles)
Shu X.O. *et al.*, 2001 [265]	1996–1998	296	359	Soy food—13–15 years, adolescence	5.4 g/day	0.51 (0.40–0.65)	Adolescent soy food intake was inversely associated with breast cancer risk

Legend: 1—for meta-analysis, the number of cases and controls were summed; 2—high degree of heterogeneity; 3—selection from maximal values presented in the analysis; a—includes case control only; b—includes case control, nested case control, prospective cohort study; c—includes survivors, deaths, recurrences; d—breast cancer survivors 6.31 years after the diagnosis—isoflavone intake and breast cancer recurrence was evaluate; Ctrl, controls; preM, premenopausal status; postM, postmenopausal status; OR, odds ratio; HR, hazard ratio; RR, risk ratio; CI, confidence interval; n.m., not mentioned; wk, week; d, day; mo, months; IF, isoflavone.

Observations from several clinical studies related to the administration of soy food to pre-menopausal and post-menopausal women or post-menopausal breast cancer survivors for 1–2 years did not significantly change the breast cancer biomarkers [274]. Earlier reports indicated that exposure to 45 mg isoflavones/day for a duration of 14 days in 48 women with benign or malignant breast lesions increased proliferation rate of the breast epithelium and up-regulated progesterone expression suggesting that short-term soy administration may increase cell proliferation [277]. The interaction of daidzein with gut bacteria leads to equol production in approximately 30% of the human population, and a direct correlation between equol production and low breast density was observed [278]. A comparison between Caucasian and Asian women indicated that the American Asian population may produce more equol compared to American Caucasian population, suggesting different metabolizing pathways [279]. Decreased cancer recurrence in a cohort of 1954 postmenopausal breast cancer survivor women was correlated with daidzein and glycetin intake compared to no isoflavone consumption [275]. Inverse correlation between the risk of breast cancer and the consumption of resveratrol from grapes, but not from wine, was observed in another study [280]. In postmenopausal women the adipose tissue produces sex steroid hormones resulting in the correlation between high adiposity and increased breast cancer risk [281]. Similarly, high adiposity was linked to low levels of sex steroid hormone binding globulin (SHBG), a protein responsible for binding of the sex steroid hormones, while up-regulation of SHBG level was associated with reduced risk of breast cancer [282]. The risk of breast cancer was associated not only with the increased production of estrogen, but also with the production of estrogen metabolites; thus, a reduced urinary ratio between 2-hydroxiestrone (2-OHE1) and 16α-hydroxiestrone (16α-OHE1), two estrogen metabolites, had been associated with increased risk of breast cancer [283]. Administration of 1 mg/day resveratrol in 40 post-menopausal women with high body mass index increased the concentration of SHBG in the plasma and that of 2-OHE1 in the urine suggesting that resveratrol may have promising effects in post-menopausal overweight women [284]. However, the side effects including diarrhea, increased serum cholesterol concentration, grade 4 elevation of liver enzymes (one subject) and grade 3 skin rashes (2 subjects) must be taken into consideration [284]. Exposure of 39 adult women with increased risk of breast cancer to 50 mg trans-resveratrol, twice/day decreased the methylation of Ras association domain family-1α (RASSF-1α), a tumor suppressor gene [285]. A study regarding consumption of EGCG (843 mg EGCG/day for 1 year) in 1075 post-menopausal women indicated that the intake of green tea extract was well tolerated [286]. Studies on the effect of EGCG in 472 patients with breast cancer revealed a decreased number of axillary lymph node metastases and a lower frequency of recurrence in pre-menopausal women with stage I-II breast cancer, while no improvement was observed in stage III breast cancer [287]. However, other data obtained with Japanese women contradict the above observations since no correlation was found between the plasma levels of the tea polyphenols and breast cancer risk [288]. Similarly, no significant association between intake of food rich in flavonols (quercetin, kaempferol and myricetin) or flavones (apigenin and luteolin) and incidence of cancers was identified in another study in which information about polyphenol consumption was obtained by food-frequency questionnaires [74]. In conclusion, the contradictory results published about the breast cancer-preventive effects of polyphenols may be related the multifactorial nature of the disease, the differences in the investigated populations and in the amount and type of dietary polyphenols consumed by the patients.

## 7. Conclusions and Further Progress

A rational life style with a reasonable level of stress, quality and moderate nutritional intake, correlated with physical exercises plays an important role in the prevention of cancer [71]. An approach similar to the aggressive chemoprevention of cardiovascular diseases might be accomplished for cancer chemoprevention as well. Measurable risk factors, such as hypercholesterolemia and hypertension, have been identified in cardiovascular disease and significant success have been achieved in eliminating them by drugs that lower cholesterol levels and reduce blood pressure [1].

Therefore, the challenge is to identify such measurable risk factors for breast cancer and remove or diminish them. Possible examples without incurring high expenses are the consumption of Asian and Mediterranean diets and eliminating or minimizing the exposure to risk factors like dietary alcohol or fat. Moreover, regular consumption of vegetables and fruits rich in polyphenols could be an alternative way in chemoprevention of cancer [2]. Cancer is frequently considered to be a chronic disease implying that chronic administration of cancer preventive compounds is required in order to inhibit carcinogenesis [1]. Again, administration of dietary polyphenols may represent one of the possible approaches. A summary of pros and cons in case of polyphenols administration in breast cancer is given in Table 6. Further *in vitro* experiments, studies using *in vivo* animal models and clinical trials are required to improve our understanding of the mechanisms of action of polyphenols for their proper application as chemo-preventive tools in breast cancer.

**Table 6:** Breast cancer: the pros and cons of polyphenols.

Pros	Cons
DNA protection by the anti-oxidant activity against carcinogens Decreased glucose uptake in cancer cells Cell cycle arrest, induction of pro-apoptotic and inhibition of anti-apoptotic proteins Modulation signaling pathways (reduced expression of plasma membrane receptors overexpressed in cancer, decreased phosphorylation of intracellular proteins) with implications in tumor growth, invasion and metastasis Generation of new classes of aromatase inhibitors based on the structure of polyphenols (flavones, isoflavones) Reduction in breast cancer risk in Chinese, Japanese women; reduction in breast cancer risk associated with high soy intake during adolescence Decreased risk of breast cancer recurrence (after daidzein intake)	So far not successful in preventing cancer in clinical trials Reduced bioavailability and stability Typically large concentrations have been used in most *in vitro* studies which are unlikely to be achieved *in vivo* The chemopreventive and therapeutic activity of polyphenols as single agents or in combination are studied in ongoing trials without final conclusions Heterogeneity in the applied doses, duration of administration, cells and animal models used in the studies
